# Supplementary material for: Specific S100 Proteins Bind Tumor Necrosis Factor and Inhibit Its Activity
Source: Int J Mol Sci. 2022 Dec 15;23(24):15956. doi: 10.3390/ijms232415956 (PMC9783754; doi:10.3390/ijms232415956)
Supplement: Supplementary file 1 [file ijms-23-15956-s001.zip › ijms-2067507-supplementary.pdf]

Supplementary Materials

Specific S100 Proteins Bind Tumor Necrosis Factor and Inhibit Its Activity

Alexey S. Kazakov[1], Marina Y. Zemskova[1,2], Gleb K. Rystsov[2], Alisa A. Vologzhannikova[1], Evgenia I. Deryusheva[1], Victoria A. Rastrygina[1], Andrey S. Sokolov[1], Maria E. Permyakova[1], Ekaterina A. Litus[1], Vladimir N. Uversky[3,*], Eugene A. Permyakov[1] and Sergei E. Permyakov[1,*]

[1] Institute for Biological Instrumentation, Pushchino Scientific Center for Biological Research of the Russian Academy of Sciences, 142290 Pushchino, Russia

[2] G.K. Skryabin Institute of Biochemistry and Physiology of Microorganisms, Pushchino Scientific Center for Biological Research of the Russian Academy of Sciences, 142290 Pushchino, Russia

[3] Department of Molecular Medicine and USF Health Byrd Alzheimer's Research Institute, Morsani College of Medicine, University of South Florida, Tampa, FL 33612, USA

* Correspondence: vuversky@usf.edu (V.N.U.); permyakov.s@gmail.com (S.E.P.); Tel.: +7-(495)-143-7740 (S.E.P.); Fax: +7-(4967)-33-05-22 (S.E.P.)

Table S1. List of the human diseases associated with TNF and S100A11 protein, according to DisGeNET database (http://www.disgenet.org). PubMed identifiers of the references confirming the protein-disease associations are indicated.

№	Disease	TNF	S100A11
1	Adenocarcinoma	8001927, 18316608, 1733439, 8227158, 18078328, 7635448, 15645141, 1733439, 1906154, 19028472, 10960444, 29028942, 17058067, 12032830, 28694750, 16467113, 19028472, 7592709, 10657935, 16313841, 10749746, 15870936	15665292, 21861103, 26544866, 15668896
2	Adenocarcinoma of lung (disorder)	30606988, 22269403, 31086598, 31540489, 7657294, 9371773, 28470556, 30942468, 29207489, 18089811, 9685865, 11053014	26544866
3	Adenoma	29286110	16327996
4	Adult glioblastoma	28604685, 31565489, 8912851, 17114809, 22591734, 26971226, 14641910, 31363754, 28317877, 27474433, 25677845, 23699535, 18670452, 10087955, 17699104, 17928957, 22318540, 26794430, 15374943, 8413882, 18709644, 26410343, 22992678, 30425514, 28351321, 28135339, 10674492, 12499252, 7566386, 16357155, 21757445, 23775076, 22266862, 23975833, 24164301, 18794145, 19836430, 25880091, 16871882, 21877214, 31059805, 10947938, 22571869	10486266, 31430050
5	Adult type dermatomyositis	10805105, 15338498, 17586554, 30367666, 25101759, 16895750, 15577608, 12485445, 19953283, 19035492, 16970978, 10399751, 12230503, 30645753, 12147628, 22913362	30684913
6	Anophthalmia and pulmonary hypoplasia	30764482, 24567526, 25213764, 30426611, 28173980, 30927045, 21109943, 28578701, 26479921	31046874
7	Bacterial endocarditis	24165416, 25299518	22319637
8	Benign prostatic hyperplasia	29441606	15668896
9	Bipolar disorder	27841086, 18515978, 28049760, 26795430, 25855618, 25855618, 25172408, 14967554, 27660275, 31214924, 28416092, 23064081, 27718369, 18515978, 17239488, 25172408, 28867391, 28445689, 31837338, 28783937	25487697
10	Bladder neoplasm	16110031, 1674182, 15533517, 23442927, 26264279, 19338536, 12966432, 14963492, 18558283, 15667866, 30066919, 22986753, 16882065, 10996723, 23727622, 25955868, 1450063, 27034531, 12966432, 16094077, 27092883	15701847
11	Breast carcinoma	10677530, 27351141, 7592751, 19553068, 9038615, 28764974, 25150312, 25591657, 19890662, 30657766, 28774312, 25175386, 20882404, 20446019, 28733879, 30442350, 29088874, 16280327, 28441391, 19087274, 15265021, 14744790, 19190336, 23174100, 15863392, 29311633, 26165253, 22941467, 28714000, 16298037, 21480222, 17047073, 25659578, 26112140, 19760502,	30662594, 25454514, 10752678

		25559835, 1764697, 22767506, 29256014, 9816237, 9419972, 31796638, 31239840, 27893337, 31730606, 31571327, 10400831, 23292149, 17283156, 28938560, 23033967, 31666931, 10880401, 20035378, 31032639, 27292433, 24301937, 31197211, 28651126, 26927216, 29202842, 29698439, 24745479, 24036252, 28127258, 1977519, 21523452, 23128672, 8661822, 28282786, 27666521, 25419573, 21490427, 31645610, 16799635, 15265702, 9157996, 16243830, 25292037, 22447108, 22750628, 28791816, 11241233, 15217507, 25813148, 28081733, 28723753, 26011589, 19446661, 23338610, 20872355, 26464679, 28627683, 11841482, 30177620, 18234964, 26331901, 27091479, 26403072, 30166590, 23991131, 19509267, 21444724, 30850950, 28369883, 14572152, 17428620, 20878356, 23143946, 23451065, 22320863, 25401416, 30728901, 24299316, 22844580, 22362301, 17516992, 23263670, 12206515, 29964331, 26553968, 21323572, 25409762, 24509793, 15999154, 25149156, 15354201, 28173834, 31382678, 30131941, 28983080, 26820653, 18409070, 29620289, 25536365, 28945218, 25332681, 19766100, 20924115, 31524251, 25704480, 29175445, 11004679, 28460534, 31454519, 30082828, 14633738, 17216494, 19632771, 26383972, 26935527, 28452243, 31659097, 30132536, 24724627, 22386367, 15286701, 25010932, 29112895, 30990165, 23053664, 20509143, 25928089, 28378740, 24598028, 25677845, 25328122, 29751615, 21476000, 27832973, 17965799	
12	Carcinogenesis	30602571, 20512382, 31775038, 20924115, 19347278, 18496150, 27053111, 22355351, 15527763, 22650374, 30096014, 27826041, 19850087, 26655880, 30893332, 23123196, 24692067, 24550071, 10671688, 25614219, 21945666, 23757449, 26722496, 22196886, 21248737, 26122654, 19528505, 25735387, 25823926, 15667484, 23512614, 29935877, 30857150, 24324738, 29981431, 21788066, 29749456, 29577454, 25102301, 31222109, 23695722, 20400476, 27977601, 27068525, 28938560, 28900035, 21237555, 24379239, 22705846, 10751892, 17145855, 28081733, 23743189, 1991983, 25354589, 28609656, 28107185, 18814840, 21631297, 18954521, 19615068, 31015764, 22025632, 27764793, 31825818, 20486865, 19823053, 12815274, 20356387, 28098875, 22707636, 21269254, 30945383, 23975421, 14578165, 22146770, 29684350, 2662193, 23292149, 28960399, 26935527, 24120915, 30218067, 25125137, 16505113, 15978325, 15474092, 25312478, 16393214, 19483191, 26112140, 22228181	29375710, 30684913, 31312359, 29568350

13	Carcinoma of bladder	30066919, 14963492, 18558283, 25955868, 19338536, 27092883, 16094077, 27034531, 16213477, 22986753, 15667866, 12966432, 23727622, 16110031, 26264279, 10996723, 16882065, 23442927, 15533517	26508026
14	Carcinoma of lung	28470556, 15014984, 29720822, 9685865, 29408308, 29379440, 21437888, 8695164, 25118107, 29345337, 28199969, 24113849, 30573629, 8978294, 24139238, 11731445, 11212267, 11773980, 31428903, 20068366, 27344406, 15864435, 25977341, 8086165, 28599466, 28676715, 28798777, 29890027, 27706681, 29080421, 30352911, 15854959, 25891208, 19564826, 30078713, 17145853, 29975933, 10945495, 22675434, 15746434, 31018714, 27998891, 26823692, 25548907, 19469656, 21699929, 27556690, 30508513, 28446126, 28099148, 30214604, 18794145, 22160576, 25562426, 28575806, 25889486, 23339680, 23174563, 20450916, 29613856, 28672902, 1680858, 29735548, 25897826, 15084385, 10423407, 30405827, 20360945, 17316570, 27738385, 31602275, 31362929, 24969564, 16476505, 26464679	21861103
15	Carcinoma of urinary bladder, superficial	1450063	26508026
16	Carcinoma, ovarian epithelial	16288025, 28848618, 8385577, 23634944, 26617713, 8166195, 19484142, 12576447, 30697874, 11406568, 17234767, 15972852, 8387543, 22095071, 24511008, 10053112, 30642295, 15047209, 21081903, 16044148, 29286125, 28044937, 28410380, 27068525, 12538625, 16505113, 8175024, 1868469, 25677845, 24676340, 20661217, 27206315, 16364318, 29802567, 26122654, 29069020	26574635
17	Cataract, Marner type	23969038	30684913
18	Cavernous hemangioma of brain	23969038	30684913
19	Cerebral infarction	29867706, 31440125, 30872008, 14593215, 29777731, 31121365, 28672984, 16173529, 26665003, 16173529, 14992821, 21562930, 27478098, 24046817, 14593215, 31032149, 31410157, 31192907, 29401724, 27585466	29844306
20	Cervical cancer	30387170, 16438713, 26191295, 23028877, 23317256, 12657115, 12142377, 22592655, 25572145, 28009429, 18650831, 25113639, 29291370, 29940817, 11600224, 15717274, 27599897, 22146770, 21670964, 23850705, 29725439, 26214423, 31825934, 17452777, 25069725, 30453149, 28669929, 22019249, 23824834, 19823053, 27039819, 25614219, 23494178, 28901517, 20117760	31695426
21	Cervical intraepithelial neoplasia	11045789, 29608425, 23363891, 19614955, 24403490, 28009429	31695426
22	Cervical squamous cell carcinoma	29146991	31695426

23	Cervix carcinoma	28901517, 25572145, 15717274, 17452777, 16438713, 23850705, 25113639, 22019249, 29940817, 29608425, 28669929, 18650831, 31825934, 11600224, 30387170, 27599897, 22146770, 26191295, 12657115, 27039819, 23028877, 19823053, 25069725, 28009429, 23317256, 30355924, 22592655, 20117760, 26214423, 21670964, 23494178, 29291370, 29725439, 12142377, 23824834, 30453149, 27145290, 22193459, 25614219	31695426
24	Childhood glioblastoma	10087955, 17928957, 27474433, 18709644, 26794430, 25880091, 22571869, 19836430, 15374943, 25677845, 30425514, 31565489, 22318540, 21757445, 28604685, 23975833, 10674492, 24164301, 17114809, 16357155, 18670452, 16871882, 22266862, 31059805, 23699535, 14641910, 28135339, 8912851, 31363754, 21877214, 17699104, 26971226, 12499252, 22591734, 26410343, 28317877, 22992678, 10947938, 8413882, 23775076, 7566386, 18794145, 28351321	10486266, 31430050
25	Childhood leukemia	26984209, 27647233, 26260845, 2790197, 24304929, 22232214, 27833035, 21875833, 22830613, 23288922, 19619938, 22762939, 30092310, 22705645, 26224647, 18205178, 26516703, 12380901, 22627572, 24488173, 9116291, 15196924, 29803792, 29326123, 27748372, 27411587, 18952893, 1699775, 10416602, 10528229, 26255115	22287738
26	Childhood osteosarcoma	20041491, 15516323, 29731867, 25335093, 31149396, 16691498, 28560396, 26097610, 27129149, 25402182, 31521127, 23999825, 22692803, 28448958, 12037623, 15695400, 30440055, 29476964, 24190483, 30093324, 29228633, 30591452, 29377600, 22355351, 2169729, 22077664	29568877
27	Choriocarcinoma	8390539, 25752285, 30591201, 17642524	22869607
28	Colorectal carcinoma	29740162, 26518902, 25174402, 28087739, 24885636, 28115787, 29695684, 30409427, 18289203, 29883699, 30999696, 12839942, 24404201, 29115450, 12717839, 25561807, 19383344, 12397645, 29552185, 28719352, 21633598, 26996193, 2662193, 27421001, 29277790, 28687755, 30509966, 29755407, 28004114, 29796912, 31707381, 31138083, 21980136, 23431386, 30509964, 21801808, 21248737, 23431386, 29238068, 8548754, 28088229, 30011858, 12478469, 31762808, 25225903, 27356745, 25380824, 30610785, 30249397, 28881611, 21573932, 9638659, 24379584, 17186550, 30591653, 27517746, 29494963, 24480808, 9891459, 23139614, 28974887, 31747664, 29801973, 28243990, 9243755, 30816524, 28575042, 27504605, 26572151, 20978325, 16937502	7889529, 16327996

29	Colorectal neoplasms	12839942, 23431386, 18433468, 25187386, 28483840, 24397824, 18289203, 24307732, 16618754, 21945666	18340452
30	Congenital chromosomal disease	15326480	30710290, 27590262
31	Conventional (clear cell) renal cell carcinoma	8894681, 30082482, 29357946, 16635513, 20566746, 25691774, 26410531, 15572731, 8483246, 1389482, 27603297, 31001636, 15814620, 22707636, 28362712, 30466107, 24788726, 8189599, 11176446, 27362805, 24530290, 28915665, 28599476	28513300, 31442606
32	Degenerative polyarthritis	29441864, 28463061, 27739057, 20491788, 26233477, 29857819, 29663159, 28692119, 20407468, 16820934, 30784197, 24657155, 31673109, 22380539, 12913922, 26409848, 27726041, 29693122, 31497200, 10229398, 28223125, 31055848, 26045761, 21352802, 30340603, 31539804, 31075239, 17604656, 31177887, 30280200, 31829201, 30557884, 17853003, 14558084, 30538044, 31228817, 25824140, 31078342, 15498798, 14687710, 30939166, 22682473, 30542428, 11966774, 28238786, 16571608, 9616438, 29484338, 22711527, 23748461, 28507472, 25193704, 30390341, 15316669, 9032818, 7622181, 29245931, 15641080, 28668088, 25461395, 30096598, 31121225, 29316707, 11508576, 29783732, 29168867, 30707846, 24269637, 31837247, 17963503, 26521731, 21858617, 25398219, 23880957, 29682561, 30844228, 24635637, 26821827, 31068673, 31434236, 23595570, 28869834, 29846433, 15517632, 10782809, 22736176, 20059371, 29138829, 27635406, 24720504, 28358372, 28849229, 23335080, 28470428, 30195882, 31396341, 31322228, 28245522, 12048293, 31210280, 30317616, 30174601, 2505790, 17216674, 30340925, 28934732, 30826358, 29891807, 31484360, 29663644, 30691048, 29247146, 28695434, 10211885, 29928231, 30087236, 31836142, 30186498, 28316377, 11352237, 12684836, 10090156, 22264405, 22939867, 24126638, 31063409, 25172660, 25252624, 31791585, 31545916, 31711940, 9461124, 16507142, 11285370, 28442110, 18438844, 29564448, 27726040, 30704030, 11728217, 31238971, 24223987, 29072705, 31497826, 31837574, 16806998, 31484919, 11920402, 17122966, 12925611, 26696755, 31735552, 26238767, 10643716, 28063227, 29549492, 31262076, 25291965, 31298308, 31282065, 31696218, 28677145, 31410125, 29581742, 25817699, 29941879, 26718307, 29163027, 27555113, 31261789, 17434489, 17968879, 30521963, 16934183	24905701, 18523305, 18523305, 28446208
33	Dermatitis, atopic	11133838, 29845271, 26419317, 27240833, 30286163, 22407167, 28530223, 28990098, 13679820, 31641232, 30400334, 29212072, 29996938, 29339444, 31437788,	18385759, 19577285

		31308264, 15560757, 30066901, 22427475, 12828754, 31059034, 31338033, 27554821, 30183461, 29977237, 31007602, 23328497, 23454147, 27289147, 21765951, 30025384, 15149503, 31172723, 29660465, 21453958, 29353040, 28833499	
34	Dermatomyositis	17586554, 12147628, 16895750, 12230503, 10805105, 17408448, 10399751, 19035492, 19953283, 15338498, 15577608, 30645753, 16970978, 30367666, 12230503, 25101759, 16895750, 19035492	30684913
35	Eczema	15149503, 13679820, 31308264, 30400334, 29660465, 29845271, 31172723, 29339444, 27554821, 30183461, 22407167, 31437788, 30286163, 31007602, 12828754, 31338033, 30066901, 11133838, 21453958, 28833499, 27240833, 26419317, 27289147, 29212072, 29023827, 30025384, 22427475, 31059034, 29428424, 29353040, 31641232, 23454147, 23328497, 27571340, 29996938, 28990098, 28530223, 29977237	19577285
36	Fibroid tumor	15474092, 31148852, 15474092, 25398968	15322223
37	Glioblastoma	31565489, 22318540, 23231075, 23975833, 23699535, 28351321, 21757445, 31125770, 29016934, 15374943, 22266862, 30863846, 21697087, 18794145, 26794430, 25358253, 23775076, 7566386, 17699104, 10947938, 25677845, 17928957, 29897522, 27474433, 26410343, 24164301, 25880091, 30718520, 14641910, 22992678, 22591734, 10087955, 16357155, 28135339, 28148846, 31059805, 10674492, 10652573, 28604685, 19836430, 16871882, 18670452, 31791385, 12499252, 29786075, 31363754, 8912851, 21877214, 29453678, 30867058, 22571869, 30425514, 28317877, 8413882, 19996278, 24244348, 18709644, 17114809, 26971226	31430050, 10486266
38	Glioblastoma multiforme	31565489, 25677845, 16357155, 27474433, 18794145, 20484045, 30867058, 28604685, 25358253, 23579274, 28148846, 19996278, 18670452, 16871882, 29016934, 24164301, 26971226, 19836430, 29453678, 31363754, 21877214, 31059805, 31125770, 21697087, 29897522, 29786075, 26410343, 12499252, 28135339, 23231075, 30049387, 30425514, 10674492, 25880091, 18709644, 23975833, 22318540, 30863846, 14641910, 28317877, 31616474, 17699104, 26794430, 30341039, 17114809, 10947938, 23775076, 30718520, 22591734, 8413882, 28351321, 15374943, 22992678, 23699535, 31791385, 24244348, 8912851, 19808964, 22266862, 7566386, 17928957, 10087955, 22571869, 21757445, 22699883	10486266, 31430050
39	Glioma	31653130, 19861406, 23640457, 28450947, 19706813, 21220502, 23975833, 22964638, 7915264, 18802741, 25622756, 22199285, 18709644, 18341587, 28103571, 30924024, 24787244, 15611130, 27393295, 15844877,	31430050

		19544410, 22318540, 24451124, 12386831, 29486949, 18708058, 23338605, 31691497, 30145468, 8910439, 16033134, 16357155, 21368892, 30049387, 31599129, 11751520, 30839135, 12957651, 26142735, 29503195, 21573233, 8421207, 21127499, 29117939	
40	Idiopathic inflammatory myopathies	15338498, 22210660, 27936488, 17586554, 11092196	30684913
41	Infective endocarditis	24165416, 25299518	22319637
42	Inflammatory disorder	24301759, 17562093, 17088647, 28411169, 25652333, 29540357, 30985099, 26352810, 30696705, 26656660, 29159717, 29914850, 29358286, 24714204, 31303399, 21604171, 28480797, 30515970, 28877989, 17483581, 16724804, 31812331, 28887124, 28676525, 29908545, 25371395	30684913
43	Influenza	24261899, 21457223, 28620671, 29548604, 29080615, 19694514, 29545454, 16253303, 15919918, 21603856, 19137067, 29157813, 19586996, 12195436, 22172537, 24641803, 30483751, 24657783, 22403659, 25103335, 31446589	19577285
44	Intrahepatic cholangiocarcinoma	28609656, 16009345, 30139338	25712376, 30182496, 29569474
45	Ischemic stroke	21219546, 30171409, 24817293, 31221990, 20493182, 31454695, 16324093, 26231680, 27586239, 26856463, 22937788, 23050663, 11737243, 19168815, 20798494, 29075930, 17452160, 30378715, 28357403, 25482248, 30484473, 28382306, 27809599, 26592822, 15036607, 30588195, 22986613	29844306
46	Laryngeal squamous cell carcinoma	23593411, 25605161	24046531
47	Leukemia	10528229, 2783672, 26260845, 22762939, 22705645, 22232214, 15196924, 9116291, 22830613, 29803792, 26516703, 18205178, 29326123, 23288922, 8043432, 10416602, 27647233, 2790197, 21875833, 28453871, 19619938, 15515013, 26255115, 1699775, 18952893, 26224647, 27833035, 12380901, 30092310, 22627572, 23071570, 20307689, 15152368, 27748372, 24304929, 24488173, 26984209, 17511776, 15515013, 27411587	22287738
48	Liver carcinoma	11749866, 27177758, 21107607, 29976395, 28063004, 26408704, 25556449, 30602571, 18030367, 14991932, 30828998, 23464434, 31710117, 23213087, 18603357, 22200181, 23811755, 27154307, 23632060, 25420786, 28115787, 15280623, 16627981, 30824840, 28367089, 21319995, 31656043, 30415242, 29444104, 24066693, 22354988, 24101445, 21469143, 19683483, 20953524, 29242605, 30051596, 30204505, 28315997, 18243134, 19910749, 30591653, 26890368, 30782845, 16237754,	19048101, 24376686, 29568350

		19795387, 21558328, 22911714, 21660448, 30844387, 28844984, 25941903, 21413021, 22200406, 24409077, 18070287, 16583461, 24751829, 25339267, 15910501, 9446586, 23751896, 31385458, 9594023, 30116311, 26683365, 21336601, 27509979, 2156928, 31268657, 18980244, 22998440, 28138696, 26323317, 28865603, 22922789, 25333816, 30809789, 25982858, 31065105, 26045814, 27022031, 26348206, 26933995, 25774942, 16820920, 28975649, 30149917, 22530834, 31807025, 20515870, 27858866, 27739418, 22682513, 22361279, 23440427, 16425355, 21928250, 23972653, 21979578, 16103092, 29090321, 26587975, 21304407, 28456632, 26672513, 26221053, 29981431, 24768182, 28579529, 25447820, 30138716, 15849810, 11059688, 22406869, 11916626, 18070287, 19780956, 25319734, 22330637, 20014456, 20819413, 30594557, 24721706, 31781194, 19376776, 23299796, 31186704, 30810331, 29506556, 31448426, 23803115, 22843047, 27121136, 30712649, 29940792, 29615720, 23141142, 31048666, 28054958, 17700059, 30178114, 15585621, 30145359, 21401328, 29720829, 19272388	
49	Malignant neoplasm of breast	26403072, 28723753, 17283156, 29311633, 11841482, 15286701, 30131941, 26935527, 27351141, 31197211, 25150312, 21480222, 21476000, 30132536, 28452243, 26927216, 25328122, 23174100, 23128672, 28938560, 24724627, 28460534, 15265702, 24745479, 28627683, 28369883, 15217507, 11431320, 9157996, 31666931, 29175445, 28127258, 28764974, 22386367, 28774312, 14633738, 16298037, 29751615, 31571327, 9038615, 24299316, 23338610, 28441391, 30850950, 25659578, 31239840, 19553068, 25292037, 9816237, 25409762, 24036252, 28983080, 31730606, 18234964, 15265021, 25591657, 1977519, 18409070, 25401416, 17216494, 10677530, 31796638, 17516992, 28945218, 26820653, 28714000, 28733879, 20035378, 28791816, 23292149, 31659097, 26464679, 30728901, 25928089, 31524251, 29202842, 31645610, 28651126, 20509143, 28173834, 8661822, 15999154, 30177620, 27282881, 27091479, 31382678, 20882404, 29698439, 25813148, 28081733, 14572152, 30082828, 16243830, 22767506, 29620289, 25332681, 29256014, 26165253, 28282786, 26383972, 15623590, 17965799, 19766100, 9199297, 21323572, 22750628, 17428620, 25010932, 30657766, 19632771, 31454519, 23451065, 26011589, 22320863, 20872355, 19760502, 25559835, 22941467, 19446661, 30990165, 21523452, 26553968, 25536365, 20878356, 23991131, 23033967, 20446019, 24598028, 29964331, 29088874,	30662594, 25454514, 10752678

		25419573, 30442350, 27893337, 23143946, 21490427, 22844580, 23263670, 20924115, 22447108, 12206515, 19087274, 30166590, 19190336, 22362301, 29112895, 27666521, 25175386, 26112140, 17047073, 23053664, 21444724, 26331901, 24301937, 25704480, 28378740, 25149156, 27292433, 27832973, 24509793, 19890662, 31032639, 24990246, 15354201	
50	Malignant neoplasm of colon and/or rectum	2662193, 18289203, 28719352, 29238068, 9638659, 23431386, 30610785, 21248737, 29883699, 27356745, 9891459, 31138083, 28687755, 29494963, 28087739, 27504605, 9243755, 29552185, 30509964, 27421001, 31747664, 19383344, 28004114, 21801808, 24404201, 28115787, 25561807, 29695684, 26572151, 21980136, 28088229, 29796912, 20978325, 21573932, 29115450, 24480808, 26518902, 24379584, 8548754, 30011858, 30509966, 12478469, 25174402, 30999696, 12397645, 12717839, 30249397, 25380824	7889529
51	Malignant neoplasm of lung	28446126, 27706681, 28798777, 29345337, 29720822, 10945495, 30405827, 23339680, 19564826, 27738385, 20068366, 29975933, 30508513, 25891208, 28672902, 29613856, 15854959, 27556690, 30214604, 28470556, 22160576, 23174563, 26464679, 31362929, 16476505, 18794145, 27998891, 22369883, 25548907, 29735548, 14587096, 25889486, 30352911, 24113849, 26823692, 22049529, 19469656, 24969564, 20112337, 24139238, 15746434, 15014984, 28099148, 21699929, 28199969, 31602275, 8086165, 23100065, 17316570, 25897826, 27344406, 8695164, 8978294, 11212267, 11773980, 17145853, 20450916, 20360945, 29080421, 10423407, 28599466, 30573629, 31018714, 30078713, 29890027, 25977341, 29379440, 29408308	21861103
52	Malignant neoplasm of ovary	26617713, 10053112, 27068525, 11406568, 8385577, 16044148, 17786312, 26122654, 16505113, 30642295, 1868469, 23634944, 12576447, 24676340, 15972852, 15047209, 8387543, 16364318, 15870936, 28044937, 30697874, 27206315, 28410380, 19484142, 22095071, 12538625, 25677845, 21081903	26574635
53	Malignant neoplasm of pancreas	16614115, 11590320, 24662747, 15781649, 29085463, 21482023, 31424683, 10469042, 9563482, 24213797, 7882388, 16211219, 21880146, 12219002, 20197756, 30011858, 17762760, 19671855, 22038233, 31464604, 29410961, 22990108, 12378118, 28440486, 11854621, 28160574, 14560157, 25213764, 19670328, 19444305	29922945, 29375710, 30850029
54	Malignant neoplasm of prostate	31718684, 29899325, 14617795, 22426894, 30349042, 18174250, 21326202, 24058525, 9917088, 29441606, 23065208, 25409762, 18196539, 12351634, 15477874, 15263792, 10815922, 27102235, 8899002, 19895686,	15668896

		24666463, 20482316, 1319775, 22355351, 15904673, 21540577, 26480897, 11289153, 27941876, 26198640, 24037407, 21237555, 15131058, 28115787, 12663665, 28223102, 29575464, 30824526, 15210850, 10751892, 29969844, 28910408, 31667686, 30027097, 15862966, 26337231, 19099590, 22955280, 12907654, 15466204, 30817944, 19846139, 15252141, 20599703, 11909978, 22923157, 17893044, 24970477, 26164758, 21097678, 26327448, 19934328, 10953159, 15545300	
55	Malignant neoplasm of soft tissue	29757258, 27884671, 19577358, 12916466	22287738
56	Malignant neoplasm of stomach	24277417, 16579840, 27373488, 12115538, 20222161, 17201881, 24249671, 28970092, 29867530, 23900678, 26504356, 1733439, 15201584, 15579481, 24789430, 19615068, 22714811, 26464679, 23975421, 28482378, 1733439, 21246243, 19604505, 15381184, 26088449, 15201584, 30094865, 18350251, 23326309, 20200422, 19626584, 15540224, 15978325, 23013936, 25548484, 29551945, 11494032, 28115787, 24072494, 19751439, 30413607, 15927855, 20699000, 24011243, 23821300, 23828749, 22459353, 31040894, 30544870, 25125137, 26069103, 19544559, 29028942, 25526211, 20512382, 16885196, 7961104, 24142527, 28699601	15138568, 30662594, 25266115
57	Malignant neoplasms	19509255, 9816329, 20446019, 7961104, 29940792, 22196886, 28708091, 25113639, 28209985, 28960310, 31747664, 17164537, 18450338, 30217305, 21476000, 28640146, 29695633, 20874489, 30546074, 22768286, 25255422, 23138871, 28938560, 14645705, 10953159, 15252138, 24122460, 27608596, 9572991, 15733831, 29085463, 9642683, 29242605, 19486922, 26107717, 26410343, 21818296, 27996200, 24301937, 28872976, 27154307, 31766230, 12894513, 29431638, 23500081, 29980532, 11983212, 28513532, 18374623, 30327303, 24072494, 16951164, 27997918, 15786692, 24056367, 17981752, 30166590, 27129149, 31040894, 20651369, 24525736, 10722927, 12478469, 29613856, 31645897, 22728043, 31400636, 31076897, 30407345, 28393234, 29668679, 1579736, 17363555, 28662099, 10757128, 21497332, 29238068, 29416724, 28639887, 30923460, 19617819, 29794474, 29552185, 29725439, 29090321, 22829912, 31534529, 22038233, 31175552, 23464434, 8231032, 22675457, 8387543, 29016879, 28677724, 23326309, 10744043, 16951242, 29288972, 15849810, 30837284, 31393373, 23339680, 26113409, 29370721, 23100065, 21327457, 22177457, 18243134, 28925992, 25849121, 18223207, 27490929, 25318697, 29490991, 24969828, 29720398, 24002210, 16452207, 22559302,	30684913, 31046874, 30773657, 29922945, 31459098, 15668896, 28513300

		24857911, 31611882, 28281970, 27531077, 28717941, 30066919, 2584719, 20978325, 31262881, 19683483, 24895231, 24587411, 10945495, 17922852, 8217820, 28463788, 7485723, 24662747, 30839135, 21573932, 25123505, 26381901, 25017974, 25704217, 21057457, 27327083, 29233887, 9583800, 25306394, 29389831, 19347278, 28705384, 29419472, 21993017, 27797827, 29039537, 30167846, 28115787, 31645676, 29475966, 22470125, 23972653, 30389698, 25015549, 19758994, 25561807, 28575042, 16832350, 18234964, 16891465, 10652573, 8473752, 28975649, 30764482, 20354842, 16199861, 25591657, 30252138, 26315998, 23647548, 28676732, 23428290, 18988868, 23415892, 11968052, 29305260, 30661394, 28628401, 22241084, 11044363, 31432544, 20646319, 30018735, 22386367, 28791816, 30307772, 30459627, 22019249, 25358405, 20564512, 26918940, 27511626, 26088082, 18990758, 16166346, 31181267, 18196539, 31790729, 30696705, 16555058, 16081933, 28446126, 21509525, 28397778, 30989732, 28225020, 28099781, 14701753, 15194046, 31817770, 30999696, 27132513, 19846961, 27927016, 11463453, 29079905, 21244577, 20388793, 11221832, 28552580, 25557131, 17071494, 16675563, 17316570, 24629840, 25174275, 16505113, 23986445, 30464207, 8917114, 27688201, 2495037, 2114216, 21993858, 16132372, 28286052, 31752383, 10751892, 29720822, 8912851, 2066357, 21904218, 22313685, 29295955, 17909069, 22752926, 25577511, 29957472, 24530290, 30628094, 24026882, 28186089, 21111705, 31524243, 25204673, 9207223, 20189297, 30569150, 30065253, 30520987, 28628031, 21699929, 28098875, 17201881, 28969419, 19418484, 30093324, 19147566, 30169594, 15509531, 12864989, 28381191, 23582782, 17848598, 28931678, 24356445, 22554523, 22592655	
58	Malignant pleural mesothelioma	10749746, 17253597, 23517112	29362358
59	Malignant tumor of cervix	25113639, 30453149, 26214423, 23850705, 12657115, 18650831, 21670964, 28669929, 28009429, 23824834, 25614219, 11600224, 17452777, 31825934, 29291370, 26191295, 23317256, 23028877, 22146770, 12142377, 16438713, 25572145, 28901517, 30387170, 15717274, 27039819, 23494178, 25069725, 20117760, 29940817, 19823053, 22592655, 29725439, 27599897, 22019249	31695426
60	Myasthenia gravis	31118027, 11574100, 9688335, 28429750, 10376950, 10199335, 24389034, 20942939, 25518708, 8982119	30684913

61	Myositis	29173693, 27578244, 24757153, 17586554, 29183282, 16970978, 29040534, 17586554, 15338498, 30604316, 10399751, 20980264, 31635041	30684913
62	Neoplasm metastasis	26858439, 26069103, 28599466, 29311633, 20332214, 31271855, 30166590, 29849497, 26165253, 28629783, 1325786, 24025362, 21326202, 28336231, 31146405, 12206515, 1389482, 27832973, 23299796, 8014015, 28969049, 29345337, 31540489, 24677135, 25010932, 28575042, 27375023, 30080276, 30989559, 21945666, 28441391, 20075981, 28482378, 29055529, 19846139, 24220622, 27941876, 31355333, 17653822, 31766230, 30244296, 27712587, 29467927, 23999825, 27511626, 25339267, 25503960, 23597429, 20197756, 29238068, 11431320, 24969828, 30082482, 28212753, 26792858, 25959126, 29115450, 27764793, 17653087, 26331901, 25102301, 24091913, 29577454, 23431386, 18764880, 26134947, 20978325, 27034531, 22855155, 18602074, 7958061, 31048666, 28275050, 30755716, 19811499, 24885636, 22320863, 1617834, 28648866, 21509525, 27556690, 16452207, 22707636, 26106605, 26112140, 21057457, 31428571, 2148875, 20087353, 24598028, 27356745, 22196886, 8032535, 17981752, 24972599, 20471161, 21573932, 23431386, 26683365, 28448958, 7485723	30662594, 31442606, 29375710, 15138568, 20388789
63	Neoplasms	26853468, 29238068, 30013362, 17510428, 24101135, 24158182, 26480897, 15604260, 8751976, 23460481, 29169725, 21442077, 26069103, 31185226, 18234964, 21097678, 25902748, 11280794, 30251217, 17653822, 27656836, 9639400, 26279161, 31324613, 26208902, 23255489, 24064911, 29608425, 8086165, 27931288, 22065587, 1499722, 30802640, 9419972, 28007595, 12434133, 16059653, 29887396, 27896636, 31786227, 28092866, 11062439, 1568183, 30103789, 12386834, 19760502, 30910714, 29109725, 12388254, 28687755, 30518097, 11221832, 12032698, 28314212, 17938269, 23817216, 9816329, 19357993, 19096444, 26768118, 28075192, 29467927, 24818725, 29460212, 8968046, 31799191, 11896437, 31803362, 22108090, 12679798, 28489607, 24969564, 12182418, 24113849, 29153059, 22086925, 15733831, 10646849, 25123505, 30665937, 17256802, 15313687, 31509504, 14508100, 30015957, 31040894, 31235854, 27793775, 8299097, 16288025, 9743290, 29124765, 11092613, 29981431, 20882532, 28891811, 16156899, 31534529, 21133714, 31726654, 31434495, 23743189, 29256014, 28881825, 29491184, 25959126, 23292149, 20197756, 30184794, 27922077, 27826041, 23674089, 23138871, 25557131, 31616474,	31430050, 15668896

		1868469, 27833035, 1905907, 24466321, 8001927, 11044363, 24489105, 15753363, 8548754, 29038485, 31213507, 10233685, 31790729, 30352261, 19543243, 9892208, 14610084, 17899369, 26983803, 30018735	
64	Non-small cell lung carcinoma	31090020, 29599338, 28446126, 29312608, 29242605, 29720822, 29613856, 30659574, 29662549, 20388802, 16476505, 21995493, 18246122, 30956278, 20423229, 16707469, 31447002, 23658645, 21769428, 30866866, 30214609, 29946223, 30930968, 27922077, 12434133, 28715437, 24969564, 29251323, 26054751, 16166445, 19665818, 16596276, 10945495, 25522273, 28599466, 28101811, 31466290, 23935876, 27016208, 30989732, 11555578, 19661294, 23012423, 30647851, 18078328, 22541966, 17620439, 30856555, 17548900, 10423407, 17639997, 25897826	21861103, 25940438
65	Osteosarcoma	29377600, 27129149, 30591452, 24190483, 29228633, 2169729, 31521127, 29476964, 28448958, 23999825, 22077664, 20041491, 15695400, 16691498, 15516323, 25335093, 30440055, 22692803, 22355351, 31149396, 26097610, 25402182, 28560396, 29731867, 30093324, 12037623	29568877
66	Osteosarcoma of bone	28448958, 31841444, 29476964, 28565880, 23999825, 25335093, 30717619, 20041491, 22355351, 16691498, 30440055, 12037623, 2169729, 22692803, 27129149, 28560396, 29228633, 25402182, 30591452, 22077664, 26097610, 31521127, 31149396, 15516323, 24190483, 29377600, 30093324, 15695400, 29731867, 31002915	29568877
67	Ovarian neoplasm	28044937, 15972852, 16908592, 16505113, 11406568, 15047209, 30642295, 12538625, 24676340, 27206315, 8385577, 10053112, 26617713, 16505113, 8387543, 19484142, 23634944, 27068525, 16044148, 21081903, 12576447, 22095071, 16364318, 26122654, 30697874	26574635
68	Pancreatic carcinoma	30011858, 15781649, 12219002, 31464604, 29085463, 20197756, 14560157, 21187523, 19444305, 25213764, 22990108, 21880146, 7882388, 10469042, 31424683, 11590320, 24213797, 19670328, 22038233, 11854621, 21482023, 12378118, 17762760, 19671855, 29410961, 7529022, 9563482, 8899800, 16614115, 24662747, 28440486, 28160574	29922945, 29375710
69	Pancreatic ductal adenocarcinoma	24091913, 25213764, 31171367, 30375637	31046874
70	Papillary thyroid carcinoma	30617967, 10438545, 29300929, 19755523, 9848725, 26289126, 1320006	25880590, 30662594, 23928665
71	Pleural effusion disorder	2518282	29362358

72	Polymyositis	16970978, 17408448, 22324944, 10805105, 30645753, 12147628, 17586554, 19953283, 15577608, 11055823, 10399751, 7979221, 15338498, 16895750	30684913
73	Primary cholangiocarcinoma of intrahepatic biliary tract	30139338	29569474, 30182496
74	Primary malignant neoplasm	26722496, 18243134, 28698503, 25854358, 29295955, 23986445, 31248045, 16132372, 25102912, 28513532, 28357602, 17363555, 25255422, 21497332, 16450333, 23339680, 29338031, 26107717, 31610150, 27154307, 28893679, 30661394, 10751892, 31645676, 8217820, 11463453, 24662747, 30999696, 28839362, 28495792, 2495037, 24969828, 23431386, 17178846, 26410343, 20388793, 16081933, 29172405, 29725439, 21111705, 20590345, 20978325, 31137684, 30724650, 23460481, 30137485, 31432544, 28705384, 22253230, 24164301, 27889231, 22228181, 12478469, 21327457, 28639101, 22707197, 25849121, 30080276, 28209985, 24857911, 16951242, 19758994, 28925992, 22116377, 22559302, 31181267, 28960310, 27996200, 30944312, 31790729, 25017974, 31752383, 19106147, 29305260, 28098875, 22313685, 17981752, 28662099, 28737121, 31766230, 22592655, 25704217, 30407345, 31437790, 29039537, 31747664, 8149602, 20874489, 20075981, 11221832, 10757128, 29490991, 15252138, 28225020, 30065253, 21244577, 19683483, 29419472, 17164537, 31509395, 25888191, 15994939, 23415892, 27569216, 29242605, 30697874, 20112337, 15150106, 20087353, 25358405, 23145797, 23794111, 20800309, 27608596, 16675563, 28820959, 28286052, 25526211, 28073079, 28639887, 18701496, 30818829, 29378114, 24530290, 24356445, 28202530, 28397014, 23138871, 28900100, 23263670, 31803890, 11295285, 2114216, 18645026, 21573932, 30252138, 18234964, 28640146, 30066919, 30341686, 26918940, 30327303, 22768286, 28708091, 28099781, 20971503, 30185628, 24501326, 8917114, 11234890, 19347278, 8101762, 29090321	31459098, 28513300, 31046874, 29922945, 15668896, 30684913, 30773657
75	Primary malignant neoplasm of lung	29735548, 20360945, 27998891, 27556690, 25977341, 17316570, 26823692, 27738385, 28446126, 30214604, 15014984, 31018714, 15746434, 30573629, 29080421, 17145853, 28798777, 27344406, 29613856, 16476505, 23174563, 30352911, 21699929, 29975933, 30508513, 19469656, 10945495, 23339680, 8695164, 29408308, 31602275, 24139238, 18794145, 11212267, 8086165, 29720822, 8978294, 22160576, 24969564, 28672902, 20450916, 29890027, 28599466, 29379440, 15854959, 11773980, 30405827, 24113849, 20068366, 28099148,	21861103

		26464679, 25897826, 27706681, 29345337, 10423407, 25889486, 28470556, 25548907, 31362929, 25891208, 28199969, 19564826, 30078713	
76	Prostate carcinoma	15252141, 8899002, 29969844, 27941876, 11568974, 21540577, 15564138, 19846139, 12907654, 20599703, 26164758, 18174250, 22923157, 10751892, 15263792, 24292881, 26480897, 24666463, 12351634, 30027097, 22426894, 12663665, 30824526, 19895686, 21270144, 29899325, 24037407, 28223102, 31667686, 27102235, 11289153, 18196539, 19099590, 23247106, 1319775, 30349042, 22955280, 11909978, 24058525, 15131058, 29441606, 10815922, 19934328, 14617795, 15862966, 24970477, 22355351, 17893044, 25409762, 27345628, 21097678, 15210850, 9917088, 23065208, 18752500, 15904673, 26327448, 30817944, 21326202, 31718684, 26198640, 15545300, 28910408, 26337231, 28115787, 29575464, 10953159, 21237555, 15466204, 20482316	15668896
77	Pterygium	15184943, 28803935, 27314284	19223989
78	Pterygium of conjunctiva and cornea	28803935, 15184943, 27314284	19223989
79	Pterygium of eye	27314284, 15184943, 28803935	19223989
80	Renal carcinoma	19164706, 24788726, 28257574	28513300
81	Renal cell carcinoma	28915665, 1404666, 8483246, 15572731, 14534724, 11176446, 12640117, 18294286, 19164706, 17475896, 1596939, 15814620, 20566746, 15614529, 8894681, 28257574, 28362712, 1389482, 8189599, 27603297, 1739130, 16635513, 22707636, 29796174, 30466107, 28599476, 24530290, 24788726	28513300
82	Rheumatoid arthritis	8216423, 20448286, 31770089, 25263964, 18797411, 25684197, 25896534, 30421069, 17599736, 28700520, 22249931, 24523570, 29254845, 28595367, 11170743, 26195802, 17343250, 31165341, 28260984, 28737121, 27779104, 28770517, 28484887, 9331953, 24673827, 29569514, 28608166, 31143951, 22258493, 22650374, 16099338, 21452922, 31659014, 23326596, 12062421, 22450443, 31179526, 21471444, 15695296, 30167920, 8507217, 30897335, 29852739, 27072520, 15517632, 29614335, 17963503, 27413250, 30079020, 26658436, 2001072, 28776300, 28288508, 31830775, 30421104, 29889832, 31105703, 30001644, 20032971, 30700574, 20664576, 21109521, 29694426, 29404725, 31065895, 17599732, 26003199, 19126414, 30778120, 11791643, 29209219, 23684916, 29082659, 28124979, 27992692, 22660986, 16331752, 28782994, 21383200, 28719435, 31779849, 16277668, 29191820, 31312201, 26453102, 30591403, 16707469, 29288410, 27307502, 28212115, 31148057, 29422529, 30828336, 26149185, 17122966,	28446208

		28775366, 27736946, 30844558, 25114059, 2026869, 30875456, 22927710, 24587984, 18461057, 21742641, 28880683, 28205331, 30526655, 29850502, 28597133, 21039421, 22121133, 22166956, 29734345, 28045034, 22264405, 3035810	
83	Sarcoma	15675481, 12916466, 27884671, 12763223, 16767912, 18065405, 30170620, 17203757, 29757258, 9794839, 12149303, 19577358	22287738
84	Schizophrenia	16252073, 16932925, 14623371, 17234379, 31211828, 18515978, 23453739, 29969604, 29074356, 30855153, 29529289, 22832610, 19939410, 16478754, 20132993, 27840055, 17171665, 15340354, 26491028, 14767724, 14767724, 29306772, 15276234, 14623371, 21127983, 31053623, 16503400, 29633506, 30627222, 15340354, 17234379, 16027740, 27886578, 12898567, 28049760, 12451465, 17171665, 31473906, 14643088, 17140746, 30268704, 12839521, 21967963, 23212700, 15927374, 17559942, 14623370, 12648734, 15927374, 30519766, 23265967, 14623370, 9270565, 28342013, 20067853, 30254506, 11244489, 29680514, 28442912, 22227290	25487697
85	Secondary malignant neoplasm of lymph node	27009073, 21573932, 12856721, 23139614, 28599466, 29575464, 26992854, 28791816, 29844685, 26464679, 20978325, 26165253, 28107185	15138568, 30182496, 31695426
86	Squamous cell carcinoma	22924547, 23870134, 17695527, 23850705, 10408389, 18206417, 22808251, 9039974, 16166279, 28885456, 18273643, 28881857, 10416616, 16202081, 8813113, 30893332, 25519014, 10651983, 24550071, 8044769, 29260411, 18206417, 11827262, 28381191, 26241630	21861103
87	Squamous cell carcinoma of esophagus	27821804, 21271217, 24324738, 26464679, 16313841, 24754231, 28050781, 28393234, 21617867, 24151445, 27731378, 30933965	15185146
88	Squamous cell carcinoma of the hypopharynx	26113409	31312359
89	Stomach carcinoma	15704154, 24277417, 19544559, 23828749, 25526211, 7961104, 18768512, 11494032, 15201584, 27556695, 15381184, 23900678, 19615068, 26088449, 23975421, 15540224, 19604505, 30413607, 26504356, 28699601, 27373488, 30094865, 26069103, 28970092, 15927855, 24011243, 12115538, 23326309, 24789430, 28482378, 24142527, 30544870, 21246243, 19751439, 25548484, 12891537, 23013936, 31040894, 20222161, 20200422, 28115787, 22714811, 16579840, 29867530, 29028942, 23821300, 19626584, 24072494, 26464679, 25125137, 27196487, 15978325, 29551945, 22459353, 15579481, 20512382, 20699000, 24249671, 17201881, 12594817, 18350251, 16885196	30662594, 25266115, 15138568

90	Stomach neoplasms	15201584, 1733439, 23016583, 18350251, 1733439, 16885196, 12730860, 15201584, 15381184, 15523691	15138568
91	Tumor cell invasion	27108701, 21246243, 20032390, 18496150, 31766230, 28465234, 28098359, 16818654, 23538445, 17516865, 24724627, 19811499, 22320863, 20087353, 23388133, 31035088, 31210280, 30961817, 31801802, 14630701, 10645003, 17021000, 27616304, 28844984, 28588103, 22767506, 22848538, 16467113, 28774312, 23431386, 26683365, 25622756, 31078266, 17673602, 28315953, 11352241, 29467899, 22124656, 23597429, 17110449, 29785588, 20103608, 21820422, 30018735, 28501501, 29633636, 24789430, 28445131, 8390539, 30661394, 18593939, 29729311, 30416660, 26272359, 31175552, 22571869, 20332214, 31540489, 27333824, 26166764, 26859114, 29467927, 26069103, 28300326, 29345337, 21543928, 30712649, 20564512, 30839135, 21544801, 26398114, 16818501, 15217903, 29294332, 19087274, 29202848, 28351321, 23460481, 22855155, 23104848, 28877735, 24064911, 22532631, 24324738, 30515818, 21905006, 26054589, 27511626, 30922443, 28242813, 23775076, 21223991, 28946560, 17609425, 26682535, 27882436, 29731867, 20460401, 23437179, 12767518, 24086682, 23699535, 28107185, 29197088, 24005829, 29022496, 25659578, 28933726, 22224671, 28693192, 29849497, 24618693, 27456070, 23975833, 23743204, 30989559, 24189042, 30451787, 29588220, 30981764, 25010932, 23674089, 24370994, 17981752, 24676340	29569474, 20388789, 30662594, 17523080, 31695426
92	Tumor progression	25664890, 30682077, 19390683, 25102301, 21828059, 29920292, 21057457, 30880468, 31766230, 23174563, 23469193, 28791816, 21980136, 25622756, 27344406, 28256185, 17142862, 11212267, 15528190, 29299026, 28275050, 20857483, 26792858, 7961104, 23932230, 24969828, 29250766, 19846139, 20878356, 30571666, 28189727, 31175552, 29175508, 8630415, 29861382, 2148874, 31540489, 27009073, 24299316, 30642295, 31695705, 10416957, 20332214, 25700355, 26165253, 31632588, 16452207	26544866, 28513300

Table S2. List of the human diseases associated with TNF and S100A11 protein, according to Open Targets Platform database (https://platform.opentargets.org/), and corresponding association scores (https://platform-docs.opentargets.org/associations#association-scores). Diseases with association scores for the both proteins exceeding 0.1 are highlighted in yellow.

№	Disease	TNF	S100A11
1	Abscess	0.016	0.007
2	Acquired idiopathic inflammatory myopathy	0.010	0.004
3	Acrodermatitis enteropathica	0.022	0.003
4	Acute hepatic failure	0.052	0.030
5	Acute kidney failure	0.089	0.004
6	Acute lymphoblastic leukemia	0.086	0.005
7	Adenocarcinoma	0.016	0.019
8	Adenoma	0.022	0.018
9	AIDS dementia	0.022	0.002
10	Alcohol dependence	0.014	0.007
11	Alcoholic liver disease	0.041	0.004
12	Allergic disease	0.081	0.036
13	Allergic rhinitis	0.099	0.040
14	Amyotrophic lateral sclerosis	0.071	0.001
15	Anaplastic large cell lymphoma	0.008	0.010
16	Arthritis	0.111	0.004
17	Asthma	0.139	0.148
18	Astrocytoma	0.020	0.015
19	Autism	0.024	0.005
20	Axial length measurement	0.073	0.013
21	Becker muscular dystrophy	0.002	0.006
22	Benign ovarian neoplasm	0.004	0.005
23	Benign prostatic hyperplasia	0.045	0.002
24	Biliary atresia	0.041	0.013
25	Bipolar disorder	0.087	0.001
26	Breast cancer	0.103	0.009
27	Breast carcinoma	0.062	0.009
28	Calf circumference measurement	0.088	0.064
29	Cancer	0.113	0.038
30	Carcinoma	0.047	0.010
31	Carcinoma, Lewis lung	0.011	0.004
32	Cerebral ischemia	0.035	0.013
33	Cerebrofacial arteriovenous metameric syndrome	0.006	0.007
34	Cervical carcinoma	0.101	0.004
35	Cervical intraepithelial neoplasia	0.012	0.003
36	Cervical squamous cell carcinoma	0.001	0.010
37	Cholangiocarcinoma	0.082	0.066
38	Chronic lymphocytic leukemia	0.045	0.003
39	Chronic obstructive pulmonary disease	0.306	0.004
40	Cirrhosis of liver	0.076	0.001

41	Colon adenoma	0.033	0.004
42	Colon carcinoma	0.071	0.004
43	Colonic neoplasm	0.026	0.004
44	Colorectal adenocarcinoma	0.015	0.009
45	Colorectal carcinoma	0.022	0.036
46	Colorectal neoplasm	0.038	0.002
47	Coronary artery disease	0.114	0.001
48	Crohn's disease	0.641	0.010
49	Decubitus ulcer	0.019	0.007
50	Dermatomyositis	0.128	0.018
51	Diabetes mellitus	0.106	0.005
52	Down syndrome	0.011	0.002
53	Duchenne muscular dystrophy	0.024	0.014
54	Eczema	0.031	0.170
55	Experimental arthritis	0.015	0.003
56	Familial adenomatous polyposis	0.008	0.004
57	Fatty liver disease	0.011	0.007
58	Follicular thyroid carcinoma	0.007	0.004
59	Gastric carcinoma	0.026	0.007
60	Glioblastoma multiforme	0.104	0.090
61	Goiter	0.001	0.002
62	Gonorrhea	0.073	0.002
63	Haim-Munk syndrome	0.008	0.004
64	Hemoglobin measurement	0.021	0.021
65	Hepatic steatosis	0.013	0.039
66	Hepatitis B virus infection	0.089	0.004
67	Hepatocellular carcinoma	0.118	0.096
68	Hyperglycemia	0.046	0.001
69	Immature platelet fraction	0.064	0.004
70	Impaired glucose tolerance	0.016	0.001
71	Infection	0.120	0.018
72	Infective endocarditis	0.014	0.007
73	Inflammatory bowel disease	0.567	0.008
74	Intrahepatic cholangiocarcinoma	0.059	0.077
75	Invasive breast ductal carcinoma	0.010	0.002
76	Ischemic disease	0.008	0.002
77	Juvenile dermatomyositis	0.025	0.015
78	Juvenile idiopathic arthritis	0.589	0.002
79	Kidney injury	0.010	0.004
80	Large cell carcinoma	0.002	0.002
81	Laryngeal carcinoma	0.009	0.006
82	Laryngeal squamous cell carcinoma	0.014	0.054
83	Leiomyoma	0.038	0.003
84	Lung adenocarcinoma	0.078	0.003
85	Lung cancer	0.267	0.001
86	Lung carcinoma	0.025	0.023

87	Lupus erythematosus	0.034	0.015
88	Lymph node metastatic carcinoma	0.010	0.013
89	Malignant pleural mesothelioma	0.018	0.074
90	MALT lymphoma	0.036	0.003
91	Mastitis	0.042	0.009
92	Medulloblastoma	0.012	0.002
93	Melanoma	0.127	0.002
94	Meningococcal infection	0.024	0.002
95	Mesothelioma	0.020	0.002
96	Metastatic malignant neoplasm	0.011	0.011
97	Metastatic melanoma	0.080	0.014
98	Myasthenia gravis	0.016	0.002
99	Myocardial infarction	0.384	0.002
100	Neoplasm	0.130	0.095
101	Neurodegenerative disease	0.018	0.001
102	Neuroendocrine neoplasm	0.042	0.003
103	Non-alcoholic fatty liver disease	0.104	0.072
104	Non-small cell lung carcinoma	0.103	0.079
105	Nonpapillary renal cell carcinoma	0.029	0.002
106	Osteoarthritis	0.524	0.018
107	Osteosarcoma	0.112	0.025
108	Ovarian cancer	0.190	0.001
109	Ovarian carcinoma	0.025	0.032
110	Pachyonychia congenita	0.033	0.024
111	Pancreatic adenocarcinoma	0.264	0.020
112	Pancreatic carcinoma	0.025	0.011
113	Pancreatic ductal adenocarcinoma	0.100	0.020
114	Papillary carcinoma	0.004	0.005
115	Papillary renal cell carcinoma	0.004	0.006
116	Papillary thyroid carcinoma	0.084	0.020
117	Parasitic infection	0.029	0.005
118	Periodontitis	0.078	0.001
119	Plasmodium falciparum malaria	0.020	0.005
120	Pleural effusion	0.049	0.063
121	Polymyositis	0.046	0.007
122	Psoriasis	0.637	0.004
123	Pulmonary arterial hypertension	0.059	0.002
124	Pulmonary hypertension	0.024	0.002
125	Relapsing-remitting multiple sclerosis	0.031	0.002
126	Renal carcinoma	0.019	0.022
127	Renal cell carcinoma	0.105	0.001
128	Rheumatoid arthritis	0.642	0.093
129	Sarcoidosis	0.367	0.002
130	Schizophrenia	0.394	0.001
131	Seasonal allergic rhinitis	0.002	0.130
132	Sepsis	0.117	0.015

133	Septic shock	0.032	0.004
134	Skin squamous cell carcinoma	0.016	0.074
135	Small cell lung carcinoma	0.024	0.011
136	Soft Tissue Neoplasm	0.014	0.001
137	Squamous cell carcinoma	0.086	0.013
138	Systemic lupus erythematosus	0.135	0.001
139	Telomere syndrome	0.016	0.001
140	Thyroid gland undifferentiated (anaplastic) carcinoma	0.023	0.002
141	Thyroid neoplasm	0.023	0.001
142	Toxoplasmosis	0.024	0.013
143	Type 2 diabetes mellitus	0.063	0.004
144	Ulcerative colitis	0.635	0.016
145	Urinary bladder carcinoma	0.054	0.024
146	Uveal melanoma	0.019	0.001

Table S3. List of the human diseases associated with TNF and S100A12 protein, according to DisGeNET database (http://www.disgenet.org). PubMed identifiers of the references confirming the protein-disease associations are indicated.

№	Disease	TNF	S100A12
1	Abdominal pain	29843500	29514815
2	Acute chest syndrome	28779156, 29845661, 29970710, 29655567, 26618233, 16098388, 22372709	28157385, 31181415
3	Acute coronary syndrome	12747595, 16098388, 16024973, 15808757, 18997459, 18997459, 17892998, 22872639, 26618233, 20981132	28157385, 31181415
4	Acute pancreatitis	27959400, 9637544, 22487393, 30144357, 31054479, 29859191, 31542607, 28300832, 29235373, 27917949, 21343291, 20396411, 29439880, 31034353, 20937337, 16886168, 30031606, 29117667, 29288273, 24371374, 29517628, 22487520, 29890468, 18580445, 29033186, 28963856, 17529903, 31518341, 28836936, 18815552, 29525967, 29393354, 15714129, 31832004, 11331456, 30106134, 30047498	29393354
5	Allergic asthma	15969671, 14987295, 29140131, 12590981, 25668892, 27240833, 12530118, 28659824, 24654313, 18053016, 16865291, 12091169	21418345, 25010197
6	Alzheimer's disease	28856541, 12446019, 28442538, 24824215, 28266558, 31443122, 19744138, 24836855, 28417590, 29714500, 17656823, 17267158, 23230229, 28982375, 15091317, 26698666, 12962917, 29969604, 16908746, 30104698, 21509504, 31076196, 15351436, 17192785, 31456514, 29375582, 31572186, 31463498, 15895461, 18396294, 30980923, 28157092, 15854776, 29568861, 29143372, 18992723, 20643858, 28943632, 29113362, 15911128, 31658056, 15895461, 21419111, 30804238, 26170148, 16516271, 28138112, 20930310, 29516540, 24998784, 27633985, 20693638, 10935448, 15501026, 25735998, 29458298, 24556805, 11121190, 11273064, 28189841, 19836613, 14745077, 31570539, 29472246, 18715507, 29899688, 31587925, 26159193, 31683445, 20110607, 9754962, 18069092, 17192785, 28947966, 22441986, 19445962, 22632257, 22580620, 15099690, 21835156, 12369958, 30542456, 15212823, 18834925, 11601494, 12116197, 18715507, 29853963, 19766542, 12782345,	28417590, 16253391

7	Androgen-insensitivity syndrome	30579843	30406853
8	Aortic aneurysm	31583036	19875725
9	Aortic aneurysm, thoracic	30088482	22818064
10	Arteriosclerosis	16224057, 18285570, 23417868, 25077563, 31486930, 28352306, 30165101, 26900933, 17276892, 22194622, 17460178, 29793330, 30235005, 18061141, 31017267, 19858416, 30844558, 18602074, 31231231, 15531779, 20644648, 17892998, 28931782, 29257205, 23249640, 18349535, 20421368, 30354204, 9863542, 27497158, 31760375, 15292354, 25323324, 25265644, 31713877, 26283334, 24012161, 30292879, 28901421, 17346438, 29062018, 29611586, 28348463, 19473660, 17347550, 9187938, 25702058, 27874938, 12753658, 28946319, 24439028, 26001207, 31817413, 18078928, 28798145, 24098801, 31208307, 30881312, 28781615, 11031205, 31727560, 25216946, 22965192, 24594319, 31294625, 21262584, 30413028, 28722106, 27102967, 28552580, 25032953, 31664319, 24905663, 30702995, 21420089, 29979389, 11728452, 31171476, 14593215, 31637885, 29250154, 24448174, 24733347, 24813629, 30426302, 11257271, 21380730, 28643478, 19803813, 15808757, 31002155, 31629987, 30176568,	31181415, 20966394, 23921255, 24389598, 26515415, 23497784, 28756107
11	Arthritis	14636987, 27619991, 15781582, 28457885, 29291937, 14871456, 7589090, 22249931, 31199978, 28771778, 16142749, 29434332, 25229347, 29452240, 27603689, 29150737, 30336303, 12234707, 28188029, 30831463, 24040061, 12198697, 24289726, 29081378, 28094754, 28438834, 12809834, 14975191, 26370562, 28442538, 30017519, 21346237, 25155522, 15743471, 20506400, 9568723, 18797411, 30185135, 30510188, 28089968, 29316707, 11840694, 17641054, 22210441, 29080328, 29743640, 11465953, 31577714, 15803022, 29145524, 26324948, 22884682, 2026869, 30747393, 23354839, 28270399, 25867281, 17934089, 17853003, 24641111, 18824582, 17195210, 28213049, 16670074, 17630192, 27269414, 1914237, 23784913, 21305533, 28118521, 30770760, 31850514, 30969024, 21452922, 24896343, 31545276,	29635517

		17516222, 30691120, 19587341, 28824645, 17514770, 30279685, 29975009, 20008919, 24420856, 30002096, 11830425, 22593232, 17416344, 28901397, 18663125, 21393509, 30261981, 10640777, 29258611, 21360496, 29859232, 29408684, 17619821, 30804932, 29681976, 8734789, 25661739, 22101928, 20032102, 27747869, 30079020, 29475857, 30783427, 29375132, 20226838, 30343885, 10395698, 31784598, 22613074, 29656315, 29868252, 29303021, 17599732, 16207331, 11096446, 29218573, 21549221,	
12	Arthritis, psoriatic	21996051, 12022360, 31672774, 15251081, 21549221, 25339124, 31719235, 29283375, 29623390, 30172115, 28823868, 20015187, 29411182, 27696735, 31277747, 16720636, 12746914, 24135300, 31112005, 31371659, 26476224, 26108918, 28010883, 29195855, 29579081, 30808625, 28772007, 29948352, 29855175, 28275260, 31652079, 29564871, 9548075, 31729183, 30916734, 29518978, 31321485, 30061880, 24594669, 30891646, 27937056, 30247726, 22480748, 21954344, 30584776, 24676037, 31521192, 30647182, 11830425, 30557124, 19732956, 15146434, 30893386, 15654990, 29846825, 12011375, 28604144, 29875003, 9326391, 28450199, 14657629, 20886249, 25980667, 28108800, 22127698, 30430682, 28386999, 28837372, 16142749, 28771778, 30742092, 28589323,	17787039
13	Arthropathy	26521731, 20136591, 30008717, 28871338, 26045761, 10782809, 25512250, 31261789, 15642137	29635517
14	Asthma	17450233, 28429138, 31780948, 10456966, 23377347, 28093718, 12091169, 27501780, 25721048, 28836545, 22376040, 18691151, 30131242, 11153913, 28831046, 19480843, 16456144, 31161819, 27001655, 14764429, 16204594, 30537802, 14681301, 16522446, 18053016, 25931357, 26722602, 21297077, 18763028, 30978295, 18088017, 31676759, 23600543, 16865291, 10390412, 18534084, 15976383, 19196817, 18088017, 30400588, 15120189, 25923882, 30590802, 21082225, 31299143, 11896460, 22947346, 28609485, 21637953, 25668892, 27808402, 23428345, 21205293, 17450233, 30738043, 18947013,	21418345

		11264706, 16517578, 18385169, 10071528, 31813561, 23855553, 30059697, 24936650, 19004142, 21307151, 16865291, 26546897, 9097957, 23573270, 30828084, 20049212, 28399677, 30193887, 20856828, 10923589, 18534084, 24588865, 17686102, 11896460, 24039878, 16728705, 22694930, 24500711,	
15	Atherosclerosis	14630568, 21262584, 28901421, 19803813, 31727560, 23095497, 15531779, 28420489, 25323324, 17460178, 29029426, 25032953, 23417868, 28576445, 22965192, 30943772, 27102967, 18349535, 12753658, 24733347, 27342458, 30292879, 28632765, 31002155, 29257205, 31059840, 11728452, 17485328, 31713877, 15808757, 19926874, 28258057, 31760375, 30235005, 30213220, 29250154, 31817413, 31231231, 20421522, 31017267, 25265644, 30426302, 26001207, 24225134, 31475703, 17346438, 28249772, 29088580, 30205791, 19473660, 30881312, 24448174, 30413028, 24439028, 19754391, 24098801, 23249640, 26218278, 31486930, 30165101, 21420089, 26459935, 17276892, 31171476, 30545708, 18061141, 16224057, 28348463, 24594319, 28946319, 18078928, 21380730, 30702995, 25077563, 22194622, 28722106, 31664319, 18285570, 26900933, 24813629, 28045034, 30066228, 26283334, 24905663, 27874938, 11031205, 30056359, 11257271, 31629987, 29979389, 30844558, 31294625, 25216946, 30041636, 31637885, 20644648, 9187938, 16487030, 28931782, 28552580, 12818408, 20421368, 17046548, 18602074, 29793330, 28798145, 28643478, 23673478, 9863542, 28352306, 16224057, 19858416, 17892998, 29401724, 29611586, 14593215, 12753658	23921255, 28756107, 20966394, 23497784, 31181415, 24389598, 18663285, 26515415
16	Bacterial sepsis	9175802, 12794406	30563403
17	Bacterial sepsis of newborn	28367457, 27960200	30420210
18	Carcinogenesis	30602571, 20512382, 31775038, 20924115, 19347278, 18496150, 27053111, 22355351, 15527763, 22650374, 30096014, 27826041, 19850087, 26655880, 30893332, 23123196, 24692067, 24550071, 10671688, 25614219, 21945666, 23757449, 26722496, 22196886, 21248737, 26122654, 19528505, 25735387, 25823926, 15667484, 23512614, 29935877,	26638166

		30857150, 24324738, 29981431, 21788066, 29749456, 29577454, 25102301, 31222109, 23695722, 20400476, 27977601, 27068525, 28938560, 28900035, 21237555, 24379239, 22705846, 10751892, 17145855, 28081733, 23743189, 1991983, 25354589, 28609656, 28107185, 18814840, 21631297, 18954521, 19615068, 31015764, 22025632, 27764793, 31825818, 20486865, 19823053, 12815274, 20356387, 28098875, 22707636, 21269254, 30945383, 23975421, 14578165, 22146770, 29684350, 2662193, 23292149, 28960399, 26935527, 24120915, 30218067, 25125137, 16505113, 15978325, 15474092, 25312478, 16393214, 19483191, 26112140, 22228181	
19	Carcinoma of lung	28470556, 15014984, 29720822, 9685865, 29408308, 29379440, 21437888, 8695164, 25118107, 29345337, 28199969, 24113849, 30573629, 8978294, 24139238, 11731445, 11212267, 11773980, 31428903, 20068366, 27344406, 15864435, 25977341, 8086165, 28599466, 28676715, 28798777, 29890027, 27706681, 29080421, 30352911, 15854959, 25891208, 19564826, 30078713, 17145853, 29975933, 10945495, 22675434, 15746434, 31018714, 27998891, 26823692, 25548907, 19469656, 21699929, 27556690, 30508513, 28446126, 28099148, 30214604, 18794145, 22160576, 25562426, 28575806, 25889486, 23339680, 23174563, 20450916, 29613856, 28672902, 1680858, 29735548, 25897826, 15084385, 10423407, 30405827, 20360945, 17316570, 27738385, 31602275, 31362929, 24969564, 16476505, 26464679	3488930, 1633879
20	Cardiovascular diseases	23624317, 16243340, 31485173, 11744409, 21420089, 29088580, 30010698, 20061995, 28625037, 20571888, 30783447, 31827125, 26757793, 18666029, 31813103, 31208307, 30925081, 16260427, 22650374, 28880739, 27798514, 31077224, 23295110, 28867452, 23024462, 17356550, 29318966, 25255422, 17705580, 22813949, 25274950, 16472051, 28256549, 28603178, 29764964, 29407876, 27477483, 17705580, 31296155, 29547707, 25175566, 15084512, 28342151, 15530917, 30081225, 16840748, 27441657, 29617535, 30279269, 29484436, 27186644, 22088456,	29212897, 28125622, 23921255, 30283343, 27914132, 29080693, 31181415

		29740313, 15531184, 29948222, 29175266, 29959484, 31037597, 30421104, 29069811, 15854959, 15808915, 31279001, 15780075, 16456090, 31220998, 30813472, 21169125, 21559831, 29112590, 28489742, 31553329, 12006640, 27796618, 30584543, 28352306, 18814905, 12058258, 30315571, 27128104, 26455386	
21	Cardiovascular morbidity	19778662	24855059
22	Central neuroblastoma	26283214, 23299796, 15065018, 21457223, 1354203, 12730964, 7906313, 30552797, 23967300, 30545920, 25503960, 15065019, 7739519, 31134519, 12615731, 15554267, 10936200, 21298033, 25890358, 28671636, 23940760, 22493319, 19954364, 7616207, 11282550	1322087
23	Cerebrovascular accident	17600229, 24500802, 31440125, 30588195, 17452160, 28844626, 29161722, 21298033, 19891854, 16473522, 30909835, 21219546, 15733973, 27955950, 27682880, 27816188, 16879054, 28751018, 21407237, 28630232, 30972077, 10552245, 14615367, 22695677, 14681891, 12565819, 28611085, 25175566, 21171972, 16879054, 31356182, 29275013, 23050663, 17109621, 10950380, 29807527, 27809686, 17600229, 11273064, 30972972, 15036607, 19168815, 31822249, 17981284, 30290827, 30120034, 21160173	29196185
24	Childhood osteosarcoma	20041491, 15516323, 29731867, 25335093, 31149396, 16691498, 28560396, 26097610, 27129149, 25402182, 31521127, 23999825, 22692803, 28448958, 12037623, 15695400, 30440055, 29476964, 24190483, 30093324, 29228633, 30591452, 29377600, 22355351, 2169729, 22077664	10209067
25	Chronic kidney diseases	28927419, 19282863, 29374468, 25585822, 28732559, 30367019, 29360163, 15600254, 22813949, 24235082, 23634661, 29659957, 21906921, 29301137, 15717635, 24012111, 31514183, 31301888, 31207307, 31673705, 29451472, 19424605, 30241461, 29369823, 31311999, 30280599, 29271576, 29048463, 31198543, 15068388, 30374604, 28489742, 30165303, 30009900, 18433704	23921255, 24855059
26	Chronic myeloproliferative disorder	30647074, 21860020, 25912019, 24463275, 26852656	29946821

27	Chronic sinusitis	26433033, 27508393, 11037831, 31794110, 28859701, 23446846	29943619
28	Cicatrix, hypertrophic	10527715, 25939875, 24962175, 22226222	25322916, 27840235
29	Complete atrioventricular block	25045829, 29054398, 20300807, 20499382, 22710188, 18190648, 15996036, 24397461, 11455194	25313445
30	Connective tissue diseases	17586554, 7731752	21740521
31	Coronary arteriosclerosis	20116810, 30053815, 23507260, 15201277, 29188498, 11689215, 25551602, 21296384, 26751459, 29940243, 17346438, 28233393, 21477635, 29734056, 31140911, 15513316, 19803813, 20571888, 28142118, 29729688, 18814905, 29330256, 31157268, 27927628, 29617535, 29686027, 28916975, 29324260, 17581788, 18063147, 30506894, 11888514, 31494339, 29399085, 29422093, 11257271, 25811595, 28739396, 25108126, 28262198, 26457306, 30633155, 22081334, 11737221, 28931782, 23357300, 23249640, 12818408, 15223716, 22691114, 27955950, 30943772, 18600307, 17516296, 11199329	28157385, 31275446, 26515415, 31794767, 19576587, 29878340
32	Coronary artery disease	28233393, 31783142, 21477635, 28739396, 29399085, 11737221, 29734056, 31140911, 19766904, 31157268, 25108126, 12972116, 25811595, 15513316, 17581788, 11357933, 17763921, 22691114, 11888514, 21296384, 27694344, 29729688, 29324260, 22088456, 18571179, 26751459, 11689215, 31494339, 23357300, 15485576, 20383745, 19644041, 29422093, 28916975, 17346438, 28414590, 12943869, 15223716, 30316971, 20493832, 30338819, 18814905, 23507260, 28262198, 15201277, 29453342, 30212581, 27955950, 17346438, 17892998, 17581788, 29686027, 25124382, 31287004, 30402858, 18063147, 18600307, 30961613, 17499233, 25551602, 29330256, 29617535, 22081334, 15339882, 15269840, 17516296, 21626471, 26884864, 30506894, 30943772, 19803813, 11257271, 30053815, 12818408	26515415, 28157385, 19576587, 29878340, 31275446, 31794767
33	Coronary heart disease	31140911, 30506894, 15223716, 20096599, 11257271, 25108126, 9502188, 29686027, 30053815, 31157268, 31324728, 12818408, 17516296, 23507260, 27957762, 29731838, 31494339, 17581788, 29896248, 18814905, 22691114, 15825968, 25551602, 29399085, 30093913, 18600307, 26751459, 19803813,	28157385, 31794767, 31275446, 29878340, 19576587, 26515415

		28262198, 11888514, 30680007, 15201277, 22081334, 21477635, 18063147, 11689215, 31746393, 25811595, 27955950, 29422093, 30943772, 23159874, 11737221, 29324260, 28916975, 29617535, 29330256, 29734056, 15513316, 17346438, 28739396, 21296384, 23357300	
34	Craniosynostosis	31794110, 31611936, 26358335, 24119601, 28859701, 23446846, 21442077, 26433033, 17578498, 21882204	29943619, 31644435
35	Crohn disease	18493210, 28747060, 28785150, 28879509, 15667501, 19895991, 29930467, 15685549, 27930408, 16534418, 27096233, 27596696, 29521042, 29333082, 27887202, 30052566, 11904678, 25501099, 19079204, 31749597, 28891095, 29875003, 29191059, 26891255, 15842589, 22960943, 28838418, 26634549, 10535868, 16611251, 25126549, 30109889, 12047261, 30558178, 30765731, 30194687, 27190304, 11502070, 24971461, 31415355, 30893421, 10344681, 12105838, 19550417, 29788272, 28417241, 15212584, 28239885, 31655907, 31336198, 18355814, 24283919, 28945638, 30430682, 29857145, 25702170, 27866429, 12428072, 29364909, 15223609, 15958080, 21296062, 22848538, 30607690, 12811429, 16641217, 30286108, 28275925, 30663072, 21887729, 28735823, 14732344, 12190096, 31006801, 31267570, 15800781, 28502609, 30056180, 26288000, 27466172, 27665176, 27895399, 15620462, 31302421, 16931032, 28752637, 28095262, 12851870, 31822667, 31279871, 23044675, 31842582, 30753560, 21829567, 30426297, 31844038, 29667146, 11313304, 11196680, 30345577, 31002701, 30897368, 31141991, 16931032, 30715262, 29872272, 30320603, 29287006, 10606965, 30665494, 9246063, 18489434, 27890854, 31089365, 31039159, 29336432, 24203631, 31574236, 29779158, 19572373, 15853915, 29673053, 28390867, 21248737, 27578800, 8913936, 30054143, 26208333, 26752469, 12809834, 29209130, 30009157, 22021194, 25637591, 28365485, 12421092, 18248655, 17565648, 26316104, 30479468, 31645276, 21402942, 29486253, 31252191, 31327402, 23774171, 29372477, 23977323,	16804393

		28426458, 25311864, 24776844, 30134234, 31814801, 29973427, 31663908, 28454853, 22860894, 17507924, 30625408, 30565265, 30205699, 25500977, 19673019, 28183530, 17255827, 31169706, 18512248, 16707968, 29293965, 29182760, 28477389, 31807751, 31605784, 28421634, 28225051, 29462395, 27933472, 28826571, 29529008, 30234366, 31703025, 31634402, 15803022, 28221250, 28837372, 26928573, 22403795, 29860529, 29788318, 12486099, 17001292, 16707469, 29920733, 29505910, 10700533, 29079905, 28039805, 30002096, 18942754, 17565648, 18422560, 10075959, 16793911, 29408806, 12789169, 29600354, 29575262, 28528528, 30773391, 31610585, 31567638, 30815825, 10210767, 28837524, 12019209, 17631138, 24018244, 12049175, 29535444, 27633266, 31642006, 27115882,	
36	Cystic fibrosis	10837369, 24603877, 17158822, 24141140, 23343370, 31428093, 27281024, 11403205, 30076968, 24062613, 22592805, 10970832, 22660986, 15790988, 21908587, 30979683, 16622779, 27397611, 24853301, 30472484, 11591888, 29233471, 9698592, 21496221, 29942313, 16622779, 16361706, 31742967, 26209880, 21659660, 11337375, 8217192, 10195071, 31262295	25754382
37	Degenerative polyarthritis	29441864, 28463061, 27739057, 20491788, 26233477, 29857819, 29663159, 28692119, 20407468, 16820934, 30784197, 24657155, 31673109, 22380539, 12913922, 26409848, 27726041, 29693122, 31497200, 10229398, 28223125, 31055848, 26045761, 21352802, 30340603, 31539804, 31075239, 17604656, 31177887, 30280200, 31829201, 30557884, 17853003, 14558084, 30538044, 31228817, 25824140, 31078342, 15498798, 14687710, 30939166, 22682473, 30542428, 11966774, 28238786, 16571608, 9616438, 29484338, 22711527, 23748461, 28507472, 25193704, 30390341, 15316669, 9032818, 7622181, 29245931, 15641080, 28668088, 25461395, 30096598, 31121225, 29316707, 11508576, 29783732, 29168867, 30707846, 24269637, 31837247, 17963503, 26521731, 21858617, 25398219, 23880957, 29682561, 30844228,	27751171, 22609404, 26400621

		24635637, 26821827, 31068673, 31434236, 23595570, 28869834, 29846433, 15517632, 10782809, 22736176, 20059371, 29138829, 27635406, 24720504, 28358372, 28849229, 23335080, 28470428, 30195882, 31396341, 31322228, 28245522, 12048293, 31210280, 30317616, 30174601, 2505790, 17216674, 30340925, 28934732, 30826358, 29891807, 31484360, 29663644, 30691048, 29247146, 28695434, 10211885, 29928231, 30087236, 31836142, 30186498, 28316377, 11352237, 12684836, 10090156, 22264405, 22939867, 24126638, 31063409, 25172660, 25252624, 31791585, 31545916, 31711940, 9461124, 16507142, 11285370, 28442110, 18438844, 29564448, 27726040, 30704030, 11728217, 31238971, 24223987, 29072705, 31497826, 31837574, 16806998, 31484919, 11920402, 17122966, 12925611, 26696755, 31735552, 26238767, 10643716, 28063227, 29549492, 31262076, 25291965, 31298308, 31282065, 31696218, 28677145, 31410125, 29581742, 25817699, 29941879, 26718307, 29163027, 27555113, 31261789, 17434489, 17968879, 30521963, 16934183	
38	Delirium	27816188, 20471115, 23385102, 30348620, 29549829	30548434
39	Diabetes	10637268, 18688044, 31834153, 30043856, 22068019, 17883332, 31502701, 31436116, 29322226, 30035378, 30673733, 30083983, 26821796, 29780390, 24424289, 29890544, 24286720, 12058258, 28463061, 14988254, 28285015, 20059422, 25684197, 21149504, 28809783, 28142150, 23159874, 16464741, 31001066, 10946317, 30054947, 31534976, 17346438, 10457150, 30660761, 16306329, 30054234, 29286405, 26704714, 28772214, 17966038, 30855435, 18348311, 31059127, 22382194, 30127248, 29456813, 29490938, 26122662, 23298660, 31685773, 24192345, 26103543, 22650374, 27955950, 30610442, 11287357, 31781724, 8608724, 28460049, 25732517, 28475641, 31177887, 12378118, 28855812, 29501767, 26419589, 29240821, 31070007, 30989829, 29414152, 25003704, 23471081, 15541048, 7682590, 23602201, 16112048, 31461798, 30279162, 30552892,	29059473, 27914132, 25754382, 12594852

		17944678, 20185810, 24934088, 24497209, 31060892, 27937047, 19118408, 29720826, 16437243, 29348480	
40	Diabetes mellitus	30552892, 31534976, 31747350, 22382194, 31177887, 24934088, 20059422, 30054234, 31502701, 31781724, 30118750, 16829307, 31167971, 23602201, 31402276, 12378118, 30550811, 29473098, 30651825, 29360163, 16437243, 23471081, 30610442, 28848152, 29367417, 17966038, 28463061, 31436116, 30127248, 16306329, 28460049, 31461798, 29568898, 29890544, 27937047, 28503994, 29490938, 28475641, 31834153, 17346438, 29348480, 28741143, 31773714, 30279162, 10672452, 24424289, 29286405, 7682590, 30083983, 23298660, 17944678, 10946317, 28855812, 14988254, 29091912, 23159874, 28142150, 30043856, 31060892, 30673733, 16464741, 30035378, 28658037, 17716775, 29720826, 18348311, 31666475, 25003704, 29322226, 20185810, 31059127, 10637268, 26419589, 11287357, 17883332, 27955950, 26821796, 26704714, 12898475, 22068019, 28809783, 29414152, 29904423, 30989829, 16622779, 24497209, 31001066, 31685773, 28000874, 25684197, 28857196, 26103543, 21149504, 24192345, 12058258, 29456813, 10457150, 24286720, 29780390, 28824543, 29501767, 31515869, 30054947, 18688044, 19118408, 26122662, 29925283, 28772214, 31070007, 16112048, 28285015, 15541048, 26686228, 30179212, 29240821, 30660761, 22650374, 10773354, 8608724, 30855435, 25732517	28652188, 12594852
41	Diabetes mellitus, insulin-dependent	16864906, 29249872, 23640034, 12366785, 19136037, 22069270, 12770796, 29257900, 28377612, 1996407, 29941029, 18247339, 12485196, 8971540, 10527399, 12270547, 17928399, 17678725, 7926295, 19530270, 29284457, 22366579, 12021142, 2572498, 24515539, 28280903, 30058243, 20185810, 23438440, 8223882, 16567828, 17130569, 19120272, 16464741, 19477545, 30945295, 10803866, 7682590, 22569251, 28426801, 8847231, 29992506, 20849900, 31054364, 29652777, 26847698, 16260427, 20686866, 21097392, 31054482, 26782543, 29206239,	28958695

		17989340, 29445851, 26114833, 27677500, 12941768, 28241993, 10566598, 12788886, 17550423, 8872171, 24677718, 17174749, 19167443, 15757867, 8093442, 31813103, 1503608, 1698309, 15627647, 11024034, 10703602, 10999832, 12021142, 8875117, 7731752, 19127455, 30497321, 28419209, 1676685, 10819246, 15149631, 15855327, 1682200, 31185481, 29312639, 8056188, 8882412, 8307785, 9174153, 18949133, 17498265, 31210557, 27890035, 23644451, 29240821, 25965561, 28640379, 12622777, 26904692, 28824543	
42	Diabetes mellitus, non-insulin-dependent	29420997, 24655058, 29945132, 30762811, 31479874, 15787661, 26095630, 17445540, 18267303, 28855812, 16677285, 10457150, 11399938, 29955954, 30021757, 29142506, 29161407, 28414180, 30142362, 29558631, 20019678, 29398906, 12818408, 21508505, 29096724, 23249316, 29687969, 29095915, 31733313, 15223990, 31450982, 28824430, 31222667, 29229313, 22869322, 12690081, 24952604, 16472051, 29551922, 31424965, 22015686, 25704106, 29032727, 30015512, 7783649, 23080424, 10405788, 9157089, 31593637, 15217754, 22382194, 18328809, 10885566, 22580620, 10528229, 31600210, 29491712, 21756351, 17445540, 19367363, 31496653, 18835531, 29605608, 21262584, 27684566, 16132956, 16050950, 23527193, 29980311, 24197696, 18249219, 20177654, 28067023, 18438653, 27421726, 31803921, 31827375, 31296192, 31485456, 26742527, 21494616, 30447052, 29896248, 31269743, 28928602, 20459604, 28393627, 23298660, 11891022, 17347550, 28105731, 12829659, 30871547, 28647512, 31545080, 12898475, 28789654, 16731844, 26883847, 26945994, 20007938, 26830076, 27822481, 29928231, 21849023, 27573980, 12788886, 31813103, 24497209, 24139907, 14988254, 28651236, 24513509, 26821796, 19955001, 10436179, 30212581, 8899294, 15541048, 23643522, 18433704, 29190710, 21289263, 29104510, 30333553, 26797519, 11891022, 27323139, 31561205, 18219773, 9562347, 17499233, 26239742, 20559450, 30638160, 29233142,	12594852, 25754382, 27914132, 29059473

		28367848, 31004310, 27832672, 28620993, 29025405, 12218380, 31355282, 11916923, 12058258, 29526533, 27349479, 22327199, 11315843, 12753658, 21249428, 25656761, 24827430, 29256014, 26782547, 28626770, 28569437, 25061733, 27317602, 24057184, 17592954, 22081334, 23900510, 16979413, 15168015, 29168915, 27649540, 27904654, 31513665, 28716139, 10878750, 12788886, 30894049, 17244513, 31363374, 25634985, 20059422, 28794498, 30917933, 24018334, 16370555, 24012111	
43	Diabetic retinopathy	17716775, 25073020, 28556866, 16979413, 29678603, 29850610, 30244600, 28767589, 27288252, 26710229, 31341796, 11640992, 18398712, 24073796, 31351870, 30535867, 28852447, 28138697, 28262614, 18413212, 26821796, 25634985, 29802904, 24655058, 26125742, 29959897, 15504978, 28969616, 28843858	31199706
44	Diarrhea	31563650, 28506572, 11160015, 30936544, 23725834, 29201570, 28823863, 20348510	27865545, 29514815
45	Encephalitis	23971732, 26816094, 28173746, 23382563, 31049785, 29373982, 30044892, 16006567, 30837977, 28284352	29408171
46	Eosinophilia	30453907, 15100677, 22909167	21418345
47	Eosinophilic disorder	15100677, 30453907, 22909167	21418345
48	Familial mediterranean fever	19346738, 16234278, 25025954, 30457980, 17950649, 16273767, 12520003, 30394352, 22736074, 22714396, 19877056, 22593232, 17057944	26486615, 30406853, 27333294, 19762364
49	Gastrointestinal inflammation	28270854	29665827
50	*Helicobacter pylori* (*H. pylori*) infection in conditions classified elsewhere and of unspecified site	12011009, 21269254, 24469254, 24249671, 25008775, 21573018, 14716517, 28130110, 29456060, 30024269, 16579840, 20699000, 11533078, 31332288, 20512382, 28746216, 21465313, 24852885, 22714811, 22459353, 17667716, 28558695, 26238217, 11705745, 15652446, 26947589, 20862490, 19751439, 18063147, 30710368, 22161133, 19276869, 26316259, 11494032, 15285015, 21502924, 17201881, 25100717, 16622749	28570542
51	Hepatitis B, chronic	29054398, 16827185, 25952099, 18221983, 19910749, 15996036, 25501140, 21847627, 26933995, 14607690, 19830555, 11455194, 20004605, 18190648, 18070287, 16824070	25313445

52	Hodgkin disease	30065253, 19131627, 30952099, 17261581, 1312061, 10953976, 8217820, 28993768, 27943169, 12030733, 24857911, 24033107, 17496310, 21439852, 17496310, 12014672, 17087739, 2386200, 11092613, 21631181, 8391931, 11418184, 15670287, 9885224, 31616638, 30857150, 10050718, 11110706	16710887, 27914132
53	Huntington disease	21439852, 21631181, 31188027, 31076648, 24360910, 27943169, 15670287	27914132, 16710887
54	Hypercholesterolemia, familial	21040915, 23131422, 22891927	30963307
55	Hyperlipoproteinemia type IIa	23131422, 21040915, 17287608	30963307
56	Hypertensive disease	15808915, 16421346, 17708628, 19282863, 27903516, 18981324, 29590257, 23071256, 29081697, 28724611, 23590298, 31756325, 31427987, 17346438, 24877626, 30610955, 29351002, 27292124, 30186428, 31646585, 31786977, 16415373, 28103274, 31823500, 12009575, 29843153, 19557004, 31042407, 31114507, 30279269, 27226618, 17708628, 10904006, 30344654, 30359461, 28595524, 12654703, 30957546, 31145298, 31063551, 28915536, 22813949, 30686655, 31485188, 29473098, 27659729, 21616679, 11357933, 30072126, 18093792, 16918352, 28233393, 31781724, 30358805, 29687969, 29668324, 28419227, 20836698, 24170558, 27847271, 22493072, 10942422, 28637815, 16202847, 18605955, 9590569, 31746626	29611244, 30818175, 28632750, 21269690
57	Idiopathic pulmonary fibrosis	29727583, 31611754, 26867691, 10934117, 8473757, 9512902, 21144722, 26709222, 30091381, 28178340, 16573560, 28629363, 19117745, 11282543, 8362967, 17205112, 15863652, 15030461	29611244
58	Impaired cognition	29867440, 29325450, 28416092, 29633506, 30593843, 31158431, 27991935, 27701469, 30348620, 30586550, 30150062, 29853963, 11517430, 29969604, 31381975, 28157092, 27697064, 29552684, 29306772, 27788929, 31835282, 30193769, 30855153, 29521253, 31030091, 25542735, 31304907, 29896269, 30026058, 30256904, 22632257, 27633985	30548434
59	Inflammation	12690081, 17660390, 18543247, 16001271, 12843254, 17530706, 12792476, 20943792, 23371441, 12606947, 21467745, 19803787, 14970111, 20167660, 26069367, 12220546,	16253391

		19324842, 21146893, 16227999, 18055234, 14988320, 17132473, 25706245, 23795810, 23348408, 11181422, 12933883, 22452660	
60	Inflammatory bowel diseases	27748629, 20693892, 30175139, 28038706, 30390672, 12428072, 30407662, 25386052, 23448791, 29518097, 25208103, 30286108, 21994466, 30884988, 30941038, 28482085, 31316377, 29740202, 12710125, 31556231, 29730603, 28505128, 28472597, 28040992, 28816757, 28523588, 28796256, 29910080, 31358760, 30745872, 28139755, 29625582, 25501099, 12421092, 29256410, 28221250, 24776844, 24120915, 28165825, 27875356, 30891036, 31535405, 29214394, 29563546, 30511938, 30811631, 28375885, 31526845, 31252191, 31457011, 27562600, 28191024, 31450748, 9246063, 28211161, 28147370, 17589947, 25775453, 31169926, 29224199, 26422515, 23723167, 29555502, 12198697, 28232073, 16707968, 27545119, 28960437, 24192118, 31326413, 28283249, 30628109, 21248737, 31536991, 29200737, 10563674, 28826571, 8913936, 31646743, 31567711, 18698679, 31504536, 26993096, 30595666, 27958281, 28120341, 14678276, 30685385, 23103332, 24850427, 31362580, 31382518, 29175527, 29382683, 21135124, 30256926, 29286110, 19230040, 29774158, 16611251, 30135331, 31340002, 23590430, 28597107, 18340362, 12811429, 27879679, 29930467, 29269245, 31211836, 8668926, 31169706, 27914136, 31772929, 31814801, 28264814, 31503216, 31191873, 28243990, 30390972, 30426297, 28039805, 31494100, 22650374, 23414308, 28934123, 30558178, 22960943, 16638698, 29746256, 31206406, 28035633, 28981653, 27665099, 30219028, 29248493, 29680064, 29763384, 29367619, 31488067, 12019209, 29383027, 29089374, 26321488, 30694569, 30888405, 30450023, 30810891, 27197066, 30773667, 28412413, 30595227, 28318043, 28238957, 27796768, 29988118, 29754190, 31098835, 30860423, 22913378, 28797042, 19710421, 29729311, 29545207, 11804840, 30818349, 25800277, 29875003, 17565649, 28711286, 29202588, 27887202, 27876682, 30209212, 28690543, 16863613,	29813107, 27787913, 21203903, 28735301, 17852869, 28708568, 28554936, 12740341, 29514815, 29665827, 27723123

		27932454, 29751976, 23274341, 16714253, 30728350, 29668916, 23211301, 16635910, 31672153, 15790356, 29663266, 20943999, 27806638, 29579933, 27528546, 29204064, 30205699, 8612993, 30521801, 28747340, 31634546, 29860529, 30939120, 29771301, 28724302, 29309905, 30327532, 25192497, 31068803, 28111380, 29370397, 24752065, 30572136, 10450517, 29358708, 27931132, 12508119, 11904678, 29176974, 31491360, 20100080, 30604370, 28823863, 29873318, 21640926, 24705148, 29210147, 31639514, 20848487,	
61	Inflammatory disorder	24301759, 17562093, 17088647, 28411169, 25652333, 29540357, 30985099, 26352810, 30696705, 26656660, 29159717, 29914850, 29358286, 24714204, 31303399, 21604171, 28480797, 30515970, 28877989, 17483581, 16724804, 31812331, 28887124, 28676525, 29908545, 25371395	19935766, 17115985
62	Juvenile arthritis	22696185, 21996051, 28223750, 29214394, 17324969, 27562034, 20596903, 29160524, 28593608, 30430682, 21205466, 11886974, 26477692, 16951943, 27849614, 30412082, 31335941, 27390133, 31823144, 24069456, 30663847, 16393767, 28597133, 19208590, 16467041, 27753221, 11975986, 31240749, 25057181, 25837015, 7495783, 26934060, 11975986, 20191493, 31646743, 29138257, 24112428, 16539828, 30185135, 24000795	15077313, 29335345, 30426025, 30225949, 27659403
63	Juvenile rheumatoid arthritis	16539828, 29214394, 16393767, 19208590, 27390133, 16951943, 8843861, 31335941, 22696185, 11886974, 16467041, 30663847, 28593608, 17324969, 31240749, 30412082, 10616003, 27562034, 19480843, 21996051, 20191493, 24069456, 28223750, 25837015, 29160524, 7495783	30426025, 27659403, 30225949, 29335345
64	Keloid	28287424, 30569148	27840235, 25322916
65	Kidney failure, acute	23566289, 29070568, 24059882, 15036247, 16595132, 12215823, 30027474, 28688148, 15627170, 26824356, 23796916, 28840836, 30973904, 30392071, 31217925, 28821332, 31497913, 23607333, 29357415, 20623750, 18460982, 29978383, 29805315, 28821873, 28410274, 30077612, 29520166, 28063928, 27788614	30810341
66	Knee pain	30134281, 28433814	27751171

67	Linear atrophy	29135875	30343334
68	Liver carcinoma	11749866, 27177758, 21107607, 29976395, 28063004, 26408704, 25556449, 30602571, 18030367, 14991932, 30828998, 23464434, 31710117, 23213087, 18603357, 22200181, 23811755, 27154307, 23632060, 25420786, 28115787, 15280623, 16627981, 30824840, 28367089, 21319995, 31656043, 30415242, 29444104, 24066693, 22354988, 24101445, 21469143, 19683483, 20953524, 29242605, 30051596, 30204505, 28315997, 18243134, 19910749, 30591653, 26890368, 30782845, 16237754, 19795387, 21558328, 22911714, 21660448, 30844387, 28844984, 25941903, 21413021, 22200406, 24409077, 18070287, 16583461, 24751829, 25339267, 15910501, 9446586, 23751896, 31385458, 9594023, 30116311, 26683365, 21336601, 27509979, 2156928, 31268657, 18980244, 22998440, 28138696, 26323317, 28865603, 22922789, 25333816, 30809789, 25982858, 31065105, 26045814, 27022031, 26348206, 26933995, 25774942, 16820920, 28975649, 30149917, 22530834, 31807025, 20515870, 27858866, 27739418, 22682513, 22361279, 23440427, 16425355, 21928250, 23972653, 21979578, 16103092, 29090321, 26587975, 21304407, 28456632, 26672513, 26221053, 29981431, 24768182, 28579529, 25447820, 30138716, 15849810, 11059688, 22406869, 11916626, 18070287, 19780956, 25319734, 22330637, 20014456, 20819413, 30594557, 24721706, 31781194, 19376776, 23299796, 31186704, 30810331	30250610
69	Lupus erythematosus, systemic	18348311, 19762398, 24865418, 29668584, 11704803, 28331294, 20954190, 18759295, 20012871, 25359881, 20140011, 30414207, 20110525, 22759859, 9777334, 25182168, 7903914, 31519970, 15932622, 30718722, 23171638, 23143392, 9182925, 30572037, 22057071, 24420856, 16095006, 19121222, 21544635, 9416858, 28367002, 20112363, 23702100, 31049024, 17665457, 29623390, 19479852, 18200501, 26107748, 22057691, 17683987, 10395102, 26582004, 11169260, 20333386, 15565953, 8895148, 26443253, 29912900, 10940893, 28848179, 10998151,	27407135, 31156624

		16391890, 15657613, 16596274, 29358664, 9543565, 1503608, 22884981, 29499037, 29063464, 7614782, 16418737, 18764842, 31024730, 11607787, 11704801, 28837372, 22913362, 25652333, 29570934, 21907587, 11958572, 29333443, 15693080, 29330524, 25762184, 11958574, 25661739, 8020556, 31734262, 20576226, 9822266, 28264814, 27943420, 1893618, 31409832, 18172443, 30729698, 24449581, 16507146, 25151985, 22820624, 12730512, 29635996, 20434119, 8813745, 15971421, 30282561, 19110660, 31081141, 27838362, 29397904, 30301439, 10703622, 10643708, 29611038, 20516030, 18001298, 30183357, 9093873, 24039598, 17665457, 18001298, 27726311, 20166876, 21499693, 22723547, 15219382, 16418737, 21163672, 29778720, 28295231	
70	Malignant neoplasms	19509255, 9816329, 20446019, 7961104, 29940792, 22196886, 28708091, 25113639, 28209985, 28960310, 31747664, 17164537, 18450338, 30217305, 21476000, 28640146, 29695633, 20874489, 30546074, 22768286, 25255422, 23138871, 28938560, 14645705, 10953159, 15252138, 24122460, 27608596, 9572991, 15733831, 29085463, 9642683, 29242605, 19486922, 26107717, 26410343, 21818296, 27996200, 24301937, 28872976, 27154307, 31766230, 12894513, 29431638, 23500081, 29980532, 11983212, 28513532, 22038233, 31175552, 23464434, 8231032, 22675457, 8387543, 29016879, 28677724, 23326309, 10744043, 16951242, 29288972, 15849810, 30837284, 31393373, 23339680, 26113409, 29370721, 23100065, 21327457, 22177457, 18243134, 28925992, 25849121, 18223207, 27490929, 25318697, 29490991, 24969828, 29720398, 24002210, 16452207, 22559302, 10945495, 17922852, 8217820, 28463788, 7485723, 24662747, 30839135, 21573932, 25123505, 26381901, 25017974, 25704217, 21057457, 27327083, 29233887, 9583800, 25306394, 29389831, 19347278, 28705384, 29419472, 21993017, 27797827, 29039537, 30167846, 28115787, 31645676, 29475966, 22470125, 23972653, 30389698, 25015549, 19758994, 25561807, 28575042,	25854354, 29881282

		16832350, 18234964, 16891465, 10652573, 8473752, 28975649, 30764482, 20354842, 16199861, 25591657, 30252138, 26315998, 23647548, 28676732, 23428290, 18988868, 23415892, 11968052, 29305260, 30661394, 28628401, 22241084, 11044363, 31432544, 20646319, 30018735, 22386367, 28791816, 30307772, 30459627, 22019249, 25358405, 20564512, 26918940, 27511626, 26088082, 18990758, 16166346, 31181267, 18196539, 31790729, 30476917, 18078328, 9338474, 28820959, 23016583, 23803115, 29462391, 24980554, 28495792, 17628160, 16373718, 25276792, 15569667, 30935038, 20075981, 18645026, 29378114, 27393295, 27641334, 19022814, 31137684, 8101762, 29172405, 12632104, 19895686, 21102426, 25854358, 29963768, 22965279, 18954521, 18381408, 16806476, 19001439, 28202530, 30049582, 27422900, 28698503, 19096444, 30696705, 16555058, 16081933, 28446126, 21509525, 28397778, 30989732, 28225020, 28099781, 14701753, 15194046, 31817770, 30999696, 27132513, 19846961, 27927016, 11463453, 29079905, 21244577, 20388793, 11221832, 28552580, 25557131, 17071494, 16675563, 17316570, 24629840, 25174275, 16505113, 23986445, 28969419, 19418484, 30093324, 19147566, 30169594, 15509531, 12864989, 28381191, 23582782, 17848598, 28931678, 24356445, 22554523, 22592655, 29338031, 26927216, 30818829, 20800309, 31555271, 17178846, 27147709, 22017428, 21187523, 21771726, 24501326, 30836096, 26214423, 7678547, 31013630, 23145797, 12145682, 12654257, 31412671, 29197678, 19540014, 19800994, 20087353, 23637280, 31509395, 31657476, 29439531, 26722496, 30185628, 20971503, 31610150, 26853468, 28687059, 23594457, 21490678, 28639101, 25987048, 28476028, 26992854, 26480897, 20682650, 23263670, 29643993, 28737121, 24404201, 23460481, 30829675, 17172826, 16114000	
71	Mucocutaneous lymph node syndrome	8387893, 23298273, 14703611, 30996002, 8996256, 29623653, 23870089, 28915665, 28108813, 18710885, 24964229, 29447181, 22100609, 26794462, 27455075, 31583766,	30447136, 20015095, 12699958

		10233729, 12783130, 29360808, 31425625, 12209524, 30988740, 28526535, 21315128, 18710885, 14985941, 29625336, 18050249, 1700705, 10902757, 24162006, 27550227, 29913529, 11328953, 29098351, 10931145, 28587409, 31261503	
72	Multiple sclerosis	10450792, 21177755, 15124762, 22641052, 17295710, 31284389, 12045717, 31785393, 28497341, 18625522, 23624563, 19244395, 17268200, 9413269, 12474981, 31681293, 26906225, 29844438, 28847534, 20535037, 24696164, 23787171, 14651518, 26267733, 9042107, 25704169, 9270565, 20082645, 28616851, 10552245, 10202987, 9345451, 31379730, 28161400, 8872171, 10871804, 9628463, 25801023, 29861718, 28131210, 12445803, 9762664, 23595117, 31726376, 31556809, 11571703, 9345390, 26062845, 31781177, 26819253, 21136171, 12568117, 18388249, 20228669, 21396892, 29210147, 27831647, 9667591, 31704454, 9094056, 18322311, 24174586, 10522904, 12084660, 17079667, 20962851, 28677863, 28939293, 9378125, 27966076, 8887999, 12120698, 27023227, 28856541, 18755820, 31559449, 9395126, 17268200, 15149631, 30206422, 9270614, 18051225, 29797117, 16900751, 28922099, 10652446, 30367633, 24475162, 1969423, 30409508, 7486863, 17439892, 11847479, 30009568, 12824709, 26844569, 11444910, 28082233, 16803996, 12175864, 20499285, 29126030, 21908876, 18348311, 28422748, 14651520, 22801493, 24790215, 29760711, 10408716, 8964905, 19246939, 29690885, 17079667, 26285213, 24024893, 15007139, 9916885, 28236206, 8550811, 10773851, 31033006, 10550297, 8624683, 10433357, 21621860, 20558237, 30143908, 30856027, 8157738, 22059991, 18573819, 25575858, 19018248, 29057962	28433998
73	Myeloproliferative disease	12036890, 26852656, 21860020, 30647074	29946821
74	Myocardial infarction	30958967, 17002900, 16310260, 21362018, 30642049, 20421368, 17981284, 31280453, 27881030, 29177025, 29258676, 18056971, 31630586, 24269648, 26498282, 14572794, 28174192, 11689215, 29434760, 30953351, 30586716, 31284167, 27487851, 31811327,	31181415

		31205590, 16714144, 29415889, 15883752, 11199329, 21380730, 26455386, 16718633, 23415700, 30068867, 30116330, 26648172, 31296837, 31666455, 27706628, 31059840, 11399901, 28599197, 16243340, 31364133, 30555342, 11113269, 16243340, 28414590, 12747595, 31218464, 28721450, 29949169	
75	Necrotizing enterocolitis in fetus or newborn	30542485, 31190184, 20716917, 31583737, 29784900, 30465999, 9044137, 29399120, 29950141, 8506954, 31125264, 28770467, 11697432	26250832, 27836422
76	Neonatal infection	28489023	30563403
77	Neoplasms	23460481, 29169725, 21442077, 26069103, 31185226, 18234964, 21097678, 25902748, 11280794, 30251217, 17653822, 27656836, 9639400, 26279161, 31324613, 26208902, 23255489, 24064911, 29608425, 8086165, 27931288, 22065587, 1499722, 30802640, 9419972, 28007595, 12434133, 16059653, 29887396, 27896636, 31786227, 28092866, 11062439, 1568183, 30103789, 12386834, 19760502, 30910714, 29109725, 12388254, 28687755, 30518097, 11221832, 12032698, 28314212, 17938269, 23817216, 9816329, 19357993, 19096444, 26768118, 28075192, 29467927, 24818725, 29460212, 8968046, 31799191, 11896437, 31803362, 22108090, 12679798, 28489607, 24969564, 12182418, 24113849, 29153059, 22086925, 15733831, 10646849, 25123505, 30665937, 17256802, 15313687, 31509504, 14508100, 30015957, 31040894, 31235854, 27793775, 8299097, 16288025, 9743290, 29124765, 11092613, 29981431, 20882532, 28891811, 11044363, 24489105, 15753363, 8548754, 29038485, 31213507, 10233685, 31790729, 30352261, 19543243, 9892208, 14610084, 17899369, 26983803, 30018735, 27556690, 19336886, 21573932, 31521127, 10945495, 24324738, 28160574, 30824549, 14978132, 15126334, 28811490, 28405578, 8473752, 27375023, 30602571, 30803506, 15374982, 18264131, 27556695, 30885361, 31659097, 11841482, 28925992, 28640146, 26106605, 31577702, 17283136, 22354145, 17594693, 30327303, 29632814, 26327448, 21771726, 25928089, 12949814, 29692616, 30945383, 28259044,	26638166, 30250610, 2557987, 29983358

23200674,	18374623,	11840265,	11004679,
24855648,	25360740,	25402182,	25677845,
29844827,	17991428,	24676340,	18508404,
31355333,	9924699,	29149434,	25973846,
31289564,	7485723,	12385006,	25113639,
27608596,	31288582,	16505113,	27993206,
20874489,	28131629,	26183926,	31271855,
20650898,	31741709,	12072546,	30285829,
25010932,	30778749,	8695814,	26124051,
15743036,	10974403,	27762493,	19090007,
29698439,	15210850,	30273860,	11594763,
8813113,	26858439,	8647234,	11836673,
25561807,	1358347,	29558152,	7812976,
21165560,	17394066,	23870134,	23531320,
28096174,	27336718,	28820959,	15293344,
9165756,	16951242,	29127109,	11067885,
30170620,	27599897,	30661394,	27068525,
26921521,	31439050,	30872117,	28969419,
27176505,	26718030,	27129149,	20087353,
29719394,	28189727,	25891416,	21536688,
19517218,	11733907,	7519125,	20308427,
12966432,	25501831,	29928491,	22293948,
15531454,	28134280,	10880020,	20332214,
11313788,	28159925,	17695527,	8215637,
23975421,	7958061,	31530042,	21480224,
21057457,	10738180,	21183948,	8380906,
10654199,	29993310,	2324115,	16438713,
7585181,	11545617,	17981752,	9573023,
26935527,	22986753,	9685865,	20580868,
31460395,	27698002,	7927889,	17560652,
12382527,	19244371,	26337231,	20509143,
21052099,	23429495,	21934654,	19843672,
31137684,	9578844,	16555058,	27254276,
29472271,	14744790,	11289151,	20603617,
11299763,	22619347,	31239343,	27832973,
15330168,	28198545,	1968360,	2556069,
21251905,	31181660,	28553282,	23016583,
28081733,	31392779,	30515264,	24269672,
24832086,	23999825,	31565489,	17653087,
23593410,	31733286,	1991983,	23299796,
24784717,	8387543,	11841484,	29197088,
22728043,	27941876,	11704860,	24678705,
31186704,	30930215,	31550511,	21945666,
30090962,	24754231,	28914439,	29062041,
29796998,	30659574,	30742987,	30341686,
22922789,	28697305,	28420351,	26426449,
17195449,	31709774,	25225903,	20035378,

		21980136, 15375575, 30310258, 19701890, 1563018, 2433337, 23774209, 12526712, 16166279, 30170303, 16178815, 20189297, 27192566, 11857407, 23178569, 18787932, 20978188	
78	Osteoporosis	28975973, 14636987, 30179212, 29203591, 31551787, 23716650, 26700816, 30001654, 28059444, 27064449, 31381476, 31364143, 15777737, 27401061, 31823500, 15587751, 28496203, 30236554, 15797957, 11196702, 31115543, 23460508, 31414225, 16410254, 28419209, 30701344, 16410254, 28121926, 15797957, 29571022, 31273943, 30387123, 22380539, 23261243, 12240899, 29535527, 30651805	17320499
79	Pancreatic neoplasm	16211219, 12378118, 18949385, 12378118	31582902
80	Parasitic diseases	20813201, 22296662	28279129
81	Periodontal diseases	24905365, 19948942, 27976595, 28833486, 27987116, 19076989, 28637991, 17763921, 23657600, 10551423, 25647273, 30158833, 30405072, 9609372, 28906456, 14723749, 9379326, 28063227, 31214897, 18355201, 29889832, 15142214, 30536725, 28282558, 12828656, 27755954, 28946056, 23368949, 16677285, 10685363, 22691114, 29740515, 18435806, 28800160, 23079986, 30321542, 20059422	29949223
82	Pneumonia	28548957, 28117761, 30126923, 23577187, 30500623, 22547270, 29139546, 31173158, 22315279, 29974374, 29516781, 29755959, 21214521, 26025100, 31012299, 31390880, 27865699, 25975517, 28204830, 30113458, 9596098, 21625544, 29769377, 31484796, 28028757, 28130082, 30317531, 28917655, 11910508, 30275916, 30478833, 31504531, 22269998, 27153924, 28035393, 17205112, 30548932, 29483931, 31318050, 28935640, 30075431, 30776316, 29872095, 28917655, 17665457, 21625544, 28511344, 29855321, 17681786, 31399022, 29889904, 25051011, 19064696, 28912506, 29432948, 28662409, 29663290, 22502799, 30599444, 28917655, 25449230, 24139879	28381820, 21418345
83	Pneumonitis	28028757, 30126923, 29139546, 29872095, 29432948, 30317531, 21625544, 28917655, 28912506, 29855321, 9596098, 25975517, 28917655, 29889904, 30176946, 28917655,	21418345

		22547270, 25051011, 30500623, 29974374, 29483931, 28035393, 27153924, 29755959, 28548957, 30075431, 30776316, 30599444, 21625544, 22315279, 28204830, 29663290, 28511344, 28117761, 29516781, 29972266, 31012299, 17205112, 28935640, 31173158, 30478833, 28662409	
84	Polycythemia vera	30703493, 24463275	29946821
85	Postoperative delirium	29137628	30548434
86	Primary biliary cirrhosis	10365808, 16938647, 10068101, 23011034, 15856047, 10515837, 19758199, 8706330, 1684248, 10453936, 12911663, 20578265, 26644386	25313445
87	Primary malignant neoplasm	26722496, 18243134, 28698503, 25854358, 29295955, 23986445, 31248045, 16132372, 25102912, 28513532, 28357602, 17363555, 25255422, 21497332, 16450333, 23339680, 29338031, 26107717, 31610150, 27154307, 28893679, 30661394, 10751892, 31645676, 8217820, 11463453, 24662747, 30999696, 28839362, 28495792, 2495037, 24969828, 23431386, 17178846, 26410343, 20388793, 16081933, 29172405, 29725439, 21111705, 20590345, 20978325, 31137684, 30724650, 23460481, 30137485, 31432544, 28705384, 22253230, 24164301, 27889231, 22228181, 12478469, 21327457, 28639101, 22707197, 25849121, 30080276, 28209985, 24857911, 16951242, 19758994, 28925992, 22116377, 22559302, 31181267, 28960310, 27996200, 30944312, 31790729, 25017974, 31752383, 19106147, 29305260, 28098875, 22313685, 17981752, 28662099, 28737121, 31766230, 22592655, 25704217, 30407345, 31437790, 29039537, 31747664, 8149602, 20874489, 20075981, 11221832, 10757128, 29490991, 15252138, 28225020, 30065253, 21244577, 19683483, 29419472, 17164537, 31509395, 25888191, 15994939, 23415892, 27569216, 29242605, 30697874, 20112337, 15150106, 20087353, 25358405, 23145797, 23794111, 20800309, 27608596, 16675563, 28820959, 28286052, 25526211, 28073079, 28639887, 18701496, 30818829, 29378114, 24530290, 24356445, 28202530, 28397014, 23138871, 28900100, 23263670, 31803890, 11295285, 2114216, 18645026, 21573932, 30252138,	25854354, 29881282

		18234964, 28640146, 30066919, 30341686, 26918940, 30327303, 22768286, 28708091, 28099781, 20971503, 30185628, 24501326, 8917114, 11234890, 19347278, 8101762, 29090321, 28552580, 30169594, 25577511, 28628031, 29967617, 15786692, 29288972, 29416724, 28931678, 25015549, 23593411, 23500081, 27531077, 23579274, 30989732, 15194046, 9583800, 18990758, 29197678, 23292149, 24107265, 16891465, 24056367, 31175552, 23582782, 21269254, 30837284, 29794474, 12864989, 30464207, 28397778, 19540014, 12632104, 31015764, 24198065, 24612949, 22898820, 8551796, 9816329, 28969419, 29431638, 29217118, 10744043, 22470125, 29475966, 27511626, 29079905, 19096444, 8528939, 29389831, 15586230, 28676732, 31076897, 18078328, 30459627, 25113639, 10946322, 24026882, 24416732, 19800994, 20564512, 28381191, 16820920, 29233887, 20189297, 19214542, 28575042, 19584265, 30829675, 17316570, 31645897, 26088082, 21993017, 24895231, 26927216, 14701753, 30105374, 28476028, 31400636, 24213797, 30839135, 8387543, 28687059, 23803115, 23637280, 16114000, 29016879, 22196886, 17106251, 29370721, 18381408, 25204673, 20651369, 11165872, 30018735, 30764482, 12894513, 16555058, 22019249, 22752926, 22038233, 28677724, 17848598, 21771726, 28587286, 28938560, 26214423, 8912851, 22675457, 27688201, 21237555, 22922338, 25959126, 31003933, 28186089, 24002210, 25987048, 25557131, 8473752, 19022814, 31412671, 29267342, 29964331, 18954521, 24122460, 29668679, 28628401, 26992854, 19147566, 28791816, 19418484, 17922852, 17909069, 30569150, 23647548, 17674351, 26516157, 19846961, 7961104, 22554523, 14645705, 17172826, 24901008, 29085463, 22965279, 28446126, 28209994, 8060337, 31834407, 21187523, , 23261452, 22728043, 29720398, 27641334, 24072494, 31524243, 23426404, 16452207, 23464434, 17628160, 31611882, 29980532, 29963768, 24629840, 31555271	
88	Primary myelofibrosis	30718771, 31297883, 29749399	29946821

89	Primary sclerosing cholangitis	31665288, 10068102, 29655972, 12612912, 28161472 11454808
90	Psoriasis	30398009, 27943384, 28818461, 28942364, 22071477 17074928, 24587411, 28638112, 15251081, 28547750, 29575262, 28940220, 29455561, 29564871, 30916734, 17553030, 12763679, 28077577, 27777066, 25692775, 29881431, 27881030, 12234707, 15186319, 25403996, 28809993, 31639514, 21317854, 16424170, 31017250, 27044681, 24211183, 28211161, 26321488, 26415694, 30654308, 21672030, 27720274, 29428424, 28421729, 24324571, 30307657, 16441627, 31719235, 28770513, 17530646, 18209089, 28477389, 15888146, 29661487, 31672774, 29023827, 30288872, 27554821, 17637826, 31457011, 25384035, 25537528, 12653732, 30972872, 29299984, 16844318, 29438576, 30321197, 28735613, 19895991, 31424708, 29035933, 30224457, 23677169, 29314201, 22113471, 27571340, 26584427, 18385757, 31384319, 30024620, 20112373, 29792084, 30016379, 20050849, 29182760, 30858782, 31017270, 24476008, 31639088, 24206262, 27524442, 30066937, 31502279, 27258623, 27538000, 26613086, 24048425, 28737221, 21085185, 19568223, 30515970, 30206447, 15086336, 26707913, 31673756, 27576147, 30030151, 29535706, 28109175, 30729703, 27670766, 24019554, 30839343, 29173945, 10844563, 31491865, 23731727, 30648836, 20931685, 31751564, 12789169, 20444206, 28643328, 22909235, 29062018, 29424306, 28899951, 17093502, 21346775, 24135300, 29751529, 27627098, 21574971, 30430682, 31646743, 30015892, 28272075, 28530223, 29389950, 30037762, 30893386, 26071216, 31240749, 29193791, 28837372, 29464730, 25946554, 28386763, 28239238, 26267733, 31101850, 29171393, 27894789, 25361234, 31775137, 28271735, 11851889, 17244977, 26563541, 30672612, 17671512, 24802997, 28258057, 29676778, 31089365, 27239673, 30662676, 23337970, 30004583, 22111980, 27793094, 23890755, 23274341, 31094242, 27043841, 28217129, 18463678, 29802736, 31012218, 27096382, 12709814, 27376466, 27774694, 24252077,

		30576759, 18296259, 27451931, 30372845, 29267456, 31485004, 30579425, 22158445, 30168849, 29938921, 28704992, 17928893, 7744320, 23946436, 29499292, 29736839, 29477734, 29737208, 30953507, 28235443, 23834030	
91	Respiration disorders	21357384, 21437888, 28859607, 20011590	25650963
92	Retinal vein occlusion	31814701	30366444
93	Rheumatoid arthritis	8216423, 20448286, 31770089, 25263964, 18797411, 25684197, 25896534, 30421069, 17599736, 28700520, 22249931, 24523570, 29254845, 28595367, 11170743, 26195802, 17343250, 31165341, 28260984, 28737121, 27779104, 28770517, 28484887, 9331953, 24673827, 29569514, 28608166, 31143951, 22258493, 22650374, 16099338, 21452922, 31659014, 23326596, 12062421, 22450443, 31179526, 21471444, 15695296, 30167920, 8507217, 30897335, 29852739, 27072520, 15517632, 29614335, 17963503, 27413250, 30079020, 26658436, 2001072, 28776300, 28288508, 31830775, 30421104, 29889832, 31105703, 30001644, 20032971, 30700574, 20664576, 21109521, 29694426, 29404725, 31065895, 17599732, 26003199, 19126414, 30778120, 11791643, 29209219, 23684916, 29082659, 28124979, 27992692, 22660986, 16331752, 28782994, 21383200, 28719435, 31779849, 16277668, 29191820, 31312201, 26453102, 30591403, 16707469, 29288410, 27307502, 28212115, 31148057, 29422529, 30828336, 26149185, 17122966, 28775366, 27736946, 30844558, 25114059, 2026869, 30875456, 22927710, 24587984, 18461057, 21742641, 28880683, 28205331, 30526655, 29850502, 28597133, 21039421, 22121133, 22166956, 29734345, 28045034, 22264405, 30358109, 28570437, 10616020, 21068383, 31261500, 1320571, 31097419, 1698773, 20053934, 29535527, 11920402, 30953327, 19822042, 20309874, 28819701, 28794503, 27255643, 16292516, 27180831, 17763852, 29895751, 20580552, 26321488, 31234900, 30506403, 16720219, 24883332, 28861167, 10817565, 28213049, 22753658, 2573586, 26814849, 23480184, 17631742, 27589926, 18634161, 31258709, 25522907, 17638513,	29885551, 31357327, 28938959, 27659403, 15973463

24040234, 31587925, 21179534, 27926504,
30323820, 20187130, 31758423, 28904316,
16277688, 16446172, 27846751, 28860618,
31663465, 28783661, 29038247, 28547815,
31457011, 29055489, 9098456, 18759306,
12730509, 30648758, 29510810, 12595628,
15361394, 30900933, 29600354, 12651614,
29693122, 31498069, 29764964, 17143972,
19389237, 22044414, 25848939, 28086756,
22685579, 31787605, 8391952, 30221485,
19411391, 28288327, 29465348, 29247146,
18381799, 16947381, 15370717, 30289538,
24286242, 26111149, 24728031, 16820934,
22196377, 30570833, 29158574, 28199970,
24382334, 17967830, 17896807, 21296193,
29667330, 24885217, 30999929, 17393389,
10857796, 30289537, 24065540, 17609389,
28692770, 28392572, 21806780, 31452079,
21420089, 29217191, 30599401, 27074847,
27567553, 28113136, 27639923, 30402698,
18381799, 29693176, 27748629, 22736176,
29149648, 27273801, 17673491, 31154413,
22039170, 29465355, 30465159, 16207322,
8582358, 31087226, 16446172, 23328930,
12847684, 19809821, 18681777, 28202745,
29197963, 10836523, 14962963, 9189051,
28488248, 27788309, 22614743, 26792343,
18438841, 25823782, 27787574, 7614782,
31096451, 25959608, 30649521, 23774171,
24558177, 21431944, 28245522, 29791439,
22884682, 31545398, 24634219, 11212177,
28239238, 19660107, 17426140, 14979058,
20080912, 30400666, 28010149, 9671990,
27619991, 18205195, 24078690, 25707877,
31010320, 28472734, 26130073, 28463788,
28316377, 1914237, 29063463, 27733572,
25941031, 21611196, 30485517, 23348744,
29623390, 9653166, 30476367, 30342345,
12960479, 30369929, 19017992, 18975340,
18824582, 16951485, 21187297, 15366373,
23335080, 25387578, 21708014, 28941039,
25460501, 27456070, 10204847, 28187781,
22246057, 26247485, 28245350, 31632872,
29872265, 19242068, 29786138, 18042642,
11838837, 27598995, 19758178, 30358982,
27943122, 29771256, 28552525, 20194454,
30552833, 30022333, 18439426, 17562093,

22992305, 27733582, 15692975, 17343250,
12566094, 29191214, 31260948, 31812161,
26560845, 31089365, 16013998, 16519794,
9717978, 26364660, 12528108, 28455580,
16564576, 22762939, 29626269, 16751383,
31028551, 31414225, 23517397, 29367417,
15899029, 18050183, 19117726, 11838837,
31842396, 9375863, 11174466, 29777367,
8163935, 26177310, 17968879, 30415451,
31208307, 31308625, 23322460, 9255106,
29394098, 19918040, 23496259, 16881868,
22055894, 31791585, 12528108, 28734661,
21304506, 11393661, 30954969, 23233654,
31240234, 10431483, 18438844, 30988705,
18427872, 28407127, 31318607, 26776603,
15104599, 11039782, 30696478, 30916218,
24574215, 29456830, 17710405, 25452308,
11229458, 22364134, 29540195, 28748515,
30587928, 30630756, 28708839, 31068444,
29067885, 10090156, 20609399, 28352145,
25348220, 20300049, 27342458, 12067756,
24574213, 20886274, 12508775, 24144456,
15248209, 31280937, 22354754, 12763679,
20472598, 9932110, 22760475, 29748156,
29352843, 28448562, 29730637, 30762811,
23295110, 27143107, 21952924, 28729087,
20112363, 19365401, 19804846, 15640148,
17562093, 17569750, 31339961, 30801951,
28409894, 20805296, 30352759, 15903021,
30232372, 28850026, 10733472, 18549443,
31670976, 30732563, 27777170, 30967248,
16820934, 31818460, 15743471, 30733963,
31523049, 17265480, 7622181, 16909270,
18797411, 29237438, 28215564, 16947419,
23217265, 23294992, 10817564, 31012365,
2001072, 28800780, 31071076, 11508576,
17824960, 19790066, 31245894, 27820799,
27749227, 27824863, 12115244, 29093151,
17314215, 24967817, 17207378, 15642135,
30941119, 12375327, 25510954, 26095630,
30304567, 16863659, 11229461, 29328500,
9117172, 19779724, 29949166, 20691091,
24489016, 29563337, 25647275, 28032317,
22480748, 18316202, 27172254, 20444206,
28849205, 29743640, 12566094, 29094311,
11269532, 12687539, 18820032, 29776906,
20039412, 28573370, 25371395, 30675683,

		28165872, 29751165, 25708025, 29329557, 27912794, 27058440, 19895991, 31036624, 28506320, 25919528, 23746537, 29471588, 11861275, 29790131, 28490617, 8751869, 20096109, 28286757, 20593016, 28619088, 26362732, 17763921, 11465707, 21777909, 29990875, 28339004, 24351865, 28871338, 29226077, 28869414, 28530020, 16142749, 29760711, 24016860, 29801753, 29224127, 7809909, 28306596, 23007924, 27434862, 18728048, 17328045, 25896535, 29391796	
94	Sepsis of the newborn	28367457, 27960200	29465314
95	Spondylarthritis	27696735, 31474599, 29869838, 25941031, 31326602, 26149736, 30941119, 17055360, 30767865, 29329443, 16947381, 29751976, 30390386, 31302784, 29517554, 28646368, 28420081, 31119895, 28893679, 29579081, 24192118, 26244882, 21640926, 28791018, 29606666, 15922688, 7622181, 12011375, 30745872, 26471338, 27490376, 31216488, 30413189, 18975340, 19493331, 16720636, 21968742, 29672807, 29806090, 27147709, 27578555, 29430880, 22614743, 29299630, 19897967, 28743358, 18166219, 22736066, 30673815, 12375327, 30206711, 31145730, 31512766, 29505325, 30824658, 29600354, 29419472, 12492251, 22972410	27659403
96	Stomach carcinoma	15704154, 24277417, 19544559, 23828749, 25526211, 7961104, 18768512, 11494032, 15201584, 27556695, 15381184, 23900678, 19615068, 26088449, 23975421, 15540224, 19604505, 30413607, 26504356, 28699601, 27373488, 30094865, 26069103, 28970092, 15927855, 24011243, 12115538, 23326309, 24789430, 28482378, 24142527, 30544870, 21246243, 19751439, 25548484, 12891537, 23013936, 31040894, 20222161, 20200422, 28115787, 22714811, 16579840, 29867530, 29028942, 23821300, 19626584, 24072494, 26464679, 25125137, 27196487, 15978325, 29551945, 22459353, 15579481, 20512382, 20699000, 24249671, 17201881, 12594817, 18350251, 16885196	29665316, 26638166
97	Synovitis, granulomatous, with uveitis and cranial neuropathies (disorder)	31006736, 31718710	30406853, 29635517

98	Systemic onset juvenile chronic arthritis	30863782, 28815559, 16393767	30406853
99	Takayasu arteritis	27771172, 27815653, 21965637, 31816847, 30010945, 31388749, 29146018, 23703343, 28778711, 24548718, 29427826, 30877209, 27564917, 30461542, 28521841, 28349250, 16720219	30594066
100	Transient ischemic attack	29777731, 11412877, 24053818, 28893572	27697435
101	Transitional cell carcinoma of bladder	18558283	25854354
102	Tuberculosis, pulmonary	29430075, 20231985, 30029895, 18931463, 14638800, 10619820, 31012357, 26345791, 29235156, 29685013, 20514989, 30295952, 26840977, 31281858, 22140472, 28249842, 8564135, 20180006, 27521485, 27404979, 20650298, 30814543, 25194676, 18931463, 12404162, 11800584, 24473766, 26774366, 27101784, 21609779, 25528189, 29325881	28110121
103	Tumor cell invasion	27108701, 21246243, 20032390, 18496150, 31766230, 28465234, 28098359, 16818654, 23538445, 17516865, 24724627, 19811499, 22320863, 20087353, 23388133, 31035088, 31210280, 30961817, 31801802, 14630701, 10645003, 17021000, 27616304, 28844984, 28588103, 22767506, 22848538, 16467113, 28774312, 23431386, 26683365, 25622756, 31078266, 17673602, 28315953, 11352241, 29467899, 22124656, 23597429, 17110449, 29785588, 20103608, 21820422, 30018735, 28501501, 29633636, 24789430, 28445131, 8390539, 30661394, 18593939, 29729311, 30416660, 26272359, 31175552, 22571869, 20332214, 31540489, 27333824, 26166764, 26859114, 29467927, 26069103, 28300326, 29345337, 21543928, 30712649, 20564512, 30839135, 21544801, 26398114, 16818501, 15217903, 29294332, 19087274, 29202848, 28351321, 23460481, 22855155, 23104848, 28877735, 24064911, 22532631, 24324738, 30515818, 21905006, 26054589, 27511626, 30922443, 28242813, 23775076, 21223991, 28946560, 17609425, 26682535, 27882436, 29731867, 20460401, 23437179, 12767518, 24086682, 23699535, 28107185, 29197088, 24005829, 29022496, 25659578, 28933726, 22224671, 28693192, 29849497, 24618693, 27456070, 23975833, 23743204, 30989559,	26638166

		24189042, 30451787, 29588220, 30981764, 25010932, 23674089, 24370994, 17981752, 24676340, 21536792	
104	Tumor progression	25664890, 30682077, 19390683, 25102301, 21828059, 29920292, 21057457, 30880468, 31766230, 23174563, 23469193, 28791816, 21980136, 25622756, 27344406, 28256185, 17142862, 11212267, 15528190, 29299026, 28275050, 20857483, 26792858, 7961104, 23932230, 24969828, 29250766, 19846139, 20878356, 30571666, 28189727, 31175552, 29175508, 8630415, 29861382, 2148874, 31540489, 27009073, 24299316, 30642295, 31695705, 10416957, 20332214, 25700355, 26165253, 31632588, 16452207	24374825, 25854354
105	Uveitis	25028373, 22736066, 31673980, 26487500, 21334264, 31021511, 8589268, 30076542, 28088912, 21357402, 30862619, 28089968, 28558009, 28528519, 19440225, 8125721, 20813201, 16581430, 19440225, 26821681, 31804623, 29455252, 30732563, 31021540, 31674159, 19657978, 10438385	29732713, 30874452
106	Vascular calcification	22437419, 21426505, 29228352, 18433704, 28789654	27914132, 20966394
107	Vascular inflammations	28286061, 31656544, 30611000, 28901414, 28116663, 30402857, 28631302, 24225134, 26964514, 29916551, 28744338, 29950823, 30186491, 30085294, 27902962, 29236702, 21614554, 23460613, 7579414, 31110200, 28386844, 26794462, 23006728	23921255, 19875725, 26515415, 31794767
108	Vasculitis	28391344, 12783130, 1696867, 27981463, 14703611, 30996002, 30375745, 10729297, 28390566, 7589090, 28705782, 31700711, 30073458, 19690440, 30158835, 27455075, 31632872, 28719435, 8455368	30533405, 12699958
109	Virus diseases	12938198, 22497974, 23785285, 30732563, 16885748, 27788058, 23889991, 24038435, 29481983, 29700762, 28650708, 21957307, 3365766, 22950839, 23895869, 15919918, 24657783, 23925283, 31333657, 9515764, 28904072, 29070690, 16081933, 26193210, 30480508, 19535454, 20874489, 30275539, 31552052, 10692445, 20921283, 28130495, 8591843, 14532286, 12559622, 28495402, 22269998, 23936072, 23148654, 28886201, 17396998, 16873280, 26657940, 24261899, 26085158, 25559996, 26950238, 24197984,	29665827

	11011080, 22718824, 31031770, 19524513, 28646884, 9440625, 27580574, 11196686, 28644900

Table S4. List of the human diseases associated with TNF and S100A12 protein, according to Open Targets Platform database (https://platform.opentargets.org/), and corresponding association scores (https://platform-docs.opentargets.org/associations#association-scores). Diseases with association scores for the both proteins exceeding 0.1 are highlighted in yellow.

№	Disease	TNF	S100A12
1	Acne	0.049	0.023
2	Acrocephalosyndactyly	0.049	0.060
3	Actinic keratosis	0.004	0.047
4	Acute coronary syndrome	0.016	0.010
5	Acute kidney failure	0.089	0.038
6	Acute lung injury	0.078	0.029
7	Acute lymphoblastic leukemia	0.086	0.004
8	Acute myeloid leukemia	0.113	0.005
9	Acute myocardial infarction	0.100	0.002
10	Acute pancreatitis	0.039	0.011
11	Acute respiratory distress syndrome	0.102	0.066
12	Adult-onset Still's disease	0.039	0.008
13	AIDS	0.057	0.019
14	Alkaline phosphatase measurement	0.066	0.007
15	Allergic disease	0.081	0.002
16	Alzheimer disease	0.139	0.017
17	Amyloidosis	0.028	0.006
18	Arthritis	0.111	0.005
19	Asthma	0.139	0.013
20	Atherosclerosis	0.043	0.027
21	Atopic eczema	0.022	0.005
22	Atrial fibrillation	0.101	0.008
23	Autoimmune disease	0.088	0.004
24	Autoimmune hepatitis	0.057	0.074
25	Autoinflammatory syndrome	0.010	0.004
26	Bacterial disease	0.050	0.005
27	Bacterial pneumonia	0.017	0.017
28	Bacterial urinary tract infection	0.020	0.001
29	Bacteriemia	0.032	0.033
30	Bartsocas-Papas syndrome	0.057	0.002
31	Behcet's syndrome	0.395	0.074
32	Benign neoplasm	0.020	0.007
33	Bladder transitional cell carcinoma	0.013	0.007
34	Blau syndrome	0.019	0.018
35	Blood protein measurement	0.285	0.043
36	Bloom syndrome	0.048	0.006
37	Brain injury	0.033	0.004
38	Breast cancer	0.103	0.015
39	Bronchopulmonary dysplasia	0.057	0.006
40	Brucellosis	0.046	0.009

41	Calf circumference measurement	0.088	0.016
42	Cancer	0.113	0.016
43	Carcinoma	0.047	0.002
44	Cardiovascular disease	0.062	0.032
45	Carotid atherosclerosis	0.017	0.018
46	Central nervous system cancer	0.005	0.015
47	Cholesteatoma	0.036	0.004
48	Cholesteryl ester measurement	0.079	0.054
49	Chorioamnionitis	0.038	0.009
50	Chronic interstitial cystitis	0.259	0.003
51	Chronic kidney disease	0.095	0.050
52	Chronic lung disease	0.009	0.001
53	Chronic lymphocytic leukemia	0.045	0.010
54	Chronic myeloproliferative disorder	0.029	0.007
55	Chronic obstructive pulmonary disease	0.306	0.059
56	Chronic periodontitis	0.045	0.018
57	Cirrhosis of liver	0.076	0.035
58	Cognitive disorder	0.005	0.001
59	Colitis	0.431	0.005
60	Colorectal adenocarcinoma	0.015	0.020
61	Colorectal carcinoma	0.022	0.001
62	Congenital rubella syndrome	0.072	0.007
63	Congestive heart failure	0.067	0.013
64	Coronary artery disease	0.114	0.041
65	COVID-19	0.347	0.018
66	Crohn's disease	0.641	0.058
67	Crohn ileitis	0.026	0.001
68	Cryopyrin-associated periodic syndrome	0.072	0.004
69	Cutaneous lupus erythematosus	0.077	0.006
70	Cutaneous mucinosis	0.008	0.002
71	Cystic fibrosis	0.100	0.009
72	Dermatomyositis	0.128	0.074
73	Diabetes mellitus	0.106	0.054
74	Diabetic neuropathy	0.021	0.001
75	Diabetic retinopathy	0.040	0.009
76	Diffuse large B-cell lymphoma	0.058	0.003
77	Digestive system neoplasm	0.010	0.003
78	Down syndrome	0.011	0.001
79	Duane retraction syndrome	0.051	0.019
80	Dysplasia	0.001	0.002
81	Emphysema	0.044	0.001
82	Encephalitis	0.025	0.001
83	Endometrial cancer	0.007	0.185
84	Endometriosis	0.094	0.003
85	Endothelial dysfunction	0.070	0.007
86	Enthesitis-related juvenile idiopathic arthritis	0.084	0.002

87	Eosinophilia	0.018	0.002
88	Eosinophilic esophagitis	0.092	0.001
89	Erythema nodosum	0.013	0.010
90	Esophageal adenocarcinoma	0.054	0.003
91	Esophageal carcinoma	0.044	0.002
92	Esophageal squamous cell carcinoma	0.103	0.001
93	Familial mediterranean fever	0.069	0.016
94	Fibromyalgia	0.048	0.001
95	Gastric carcinoma	0.026	0.010
96	Gastroenteritis	0.010	0.003
97	Gastrointestinal disease	0.028	0.005
98	Generalized pustular psoriasis	0.282	0.002
99	Gingivitis	0.028	0.002
100	Glomerulonephritis	0.023	0.018
101	Gonorrhea	0.073	0.024
102	Gout	0.378	0.002
103	Graft versus host disease	0.466	0.001
104	Head and neck squamous cell carcinoma	0.040	0.007
105	Heart failure	0.058	0.007
106	Helminthiasis	0.026	0.001
107	Hematoma	0.036	0.002
108	Hepatocellular carcinoma	0.118	0.043
109	Hip fracture	0.038	0.010
110	HIV infection	0.043	0.002
111	Huntington disease	0.053	0.018
112	Hypercholesterolemia	0.013	0.007
113	Hyperglycemia	0.046	0.013
114	Hyperlipidemia	0.027	0.005
115	Hypoalbuminemia	0.010	0.009
116	Idiopathic pulmonary fibrosis	0.029	0.003
117	Immature platelet fraction	0.064	0.002
118	Immune system disease	0.547	0.001
119	Infection	0.120	0.041
120	Infectious disease	0.027	0.009
121	Infertility	0.039	0.021
122	Inflammatory bowel disease	0.567	0.103
123	Insulin resistance	0.114	0.005
124	Interstitial lung disease	0.102	0.075
125	Iintestinal disease	0.014	0.001
126	Intracerebral hemorrhage	0.070	0.010
127	Irritable bowel syndrome	0.044	0.093
128	Juvenile idiopathic arthritis	0.589	0.033
129	Kidney disease	0.023	0.043
130	Leishmaniasis	0.051	0.018
131	Lepromatous leprosy	0.013	0.002
132	Leprosy	0.072	0.037

133	Liver disease	0.010	0.011
134	Lung adenocarcinoma	0.078	0.017
135	Lung carcinoma	0.025	0.033
136	Lung disease	0.019	0.001
137	Lupus nephritis	0.045	0.008
138	Lyme disease	0.018	0.006
139	Macroglobulinemia	0.001	0.003
140	Major depressive disorder	0.106	0.001
141	Mal de Meleda	0.014	0.001
142	MALT lymphoma	0.036	0.006
143	Mastitis	0.042	0.023
144	Melanoma	0.127	0.007
145	Meningococcal infection	0.024	0.030
146	Metabolic syndrome	0.103	0.001
147	Moebius syndrome	0.018	0.010
148	Muckle-Wells syndrome	0.004	0.011
149	Mucocutaneous lymph node syndrome	0.418	0.085
150	Mucositis	0.023	0.001
151	Mucous membrane pemphigoid	0.079	0.005
152	Multiple sclerosis	0.050	0.007
153	Mycobacterium infection	0.033	0.009
154	Myeloid leukemia	0.025	0.001
155	Myeloid sarcoma	0.102	0.024
156	Myeloproliferative disorder	0.030	0.007
157	Myocardial infarction	0.384	0.002
158	Myocardial ischemia	0.085	0.002
159	Necrotizing enterocolitis	0.070	0.045
160	Neonatal diabetes mellitus	0.028	0.004
161	Neoplasm	0.130	0.071
162	Neoplastic disease or syndrome	0.004	0.004
163	Nephritis	0.019	0.004
164	Nervous system disease	0.027	0.001
165	Neu-Laxova syndrome	0.025	0.004
166	Neuroendocrine neoplasm	0.042	0.005
167	Newborn respiratory distress syndrome	0.018	0.010
168	Non-small cell lung carcinoma	0.103	0.033
169	Nosocomial infection	0.010	0.002
170	Obesity	0.100	0.004
171	Obstructive sleep apnea	0.112	0.083
172	Oncostatin-M measurement	0.090	0.006
173	Oral squamous cell carcinoma	0.109	0.002
174	Oropharynx squamous cell carcinoma	0.008	0.008
175	Osteoarthritis	0.524	0.012
176	Osteoarthritis, knee	0.394	0.009
177	Osteoporosis	0.066	0.002
178	Osteosarcoma	0.112	0.016

179	Otitis media	0.055	0.009
180	Pallister-Hall syndrome	0.096	0.002
181	Pancolitis	0.013	0.003
182	Pancreatic neoplasm	0.098	0.013
183	Pancreatitis	0.056	0.002
184	Papillary thyroid carcinoma	0.084	0.076
185	Parasitic infection	0.029	0.001
186	Peri-implantitis	0.042	0.001
187	Periodontal disease	0.057	0.011
188	Periodontitis	0.078	0.002
189	Peripheral arterial disease	0.028	0.071
190	Peripheral vascular disease	0.005	0.001
191	Peritonitis	0.037	0.007
192	Pick disease	0.015	0.002
193	Plasmodium falciparum malaria	0.020	0.004
194	Pneumococcal meningitis	0.020	0.001
195	Pneumonia	0.280	0.023
196	Pneumonitis	0.071	0.004
197	Polyarticular juvenile idiopathic arthritis	0.005	0.001
198	Polycythemia vera	0.042	0.004
199	Posterior leukoencephalopathy syndrome	0.076	0.006
200	Prediabetes syndrome	0.033	0.004
201	Primary biliary cirrhosis	0.057	0.031
202	Proctitis	0.011	0.003
203	Prostate adenocarcinoma	0.008	0.003
204	Psoriasis	0.637	0.090
205	Psoriasis vulgaris	0.375	0.007
206	Psoriatic arthritis	0.693	0.015
207	Pulmonary tuberculosis	0.104	0.045
208	Relapsing-remitting multiple sclerosis	0.031	0.005
209	Renal insufficiency	0.039	0.002
210	Respiratory syncytial virus infection	0.048	0.004
211	Respiratory system disease	0.021	0.008
212	Rheumatic disease	0.026	0.001
213	Rheumatoid arthritis	0.642	0.072
214	Rosacea	0.011	0.028
215	SAPHO syndrome	0.047	0.001
216	Sarcoidosis	0.367	0.002
217	Sclerosing cholangitis	0.035	0.001
218	Sepsis	0.117	0.052
219	Septic shock	0.032	0.025
220	Serous cystadenoma	0.004	0.004
221	Serum lipopolysaccharide activity	0.052	0.003
222	Severe acute respiratory syndrome	0.016	0.007
223	Sjogren syndrome	0.083	0.015
224	Skin infection	0.042	0.001

225	Spindle cell hemangioma	0.021	0.002
226	Spondyloarthropathy	0.582	0.001
227	Squamous cell carcinoma	0.086	0.047
228	Stomach disease	0.014	0.004
229	Synovitis	0.082	0.005
230	Systemic juvenile idiopathic arthritis	0.063	0.055
231	Systemic lupus erythematosus	0.135	0.063
232	Systemic scleroderma	0.032	0.015
233	Tarsal-carpal coalition syndrome	0.003	0.002
234	Temporal arteritis	0.282	0.005
235	Thanatophoric dysplasia	0.019	0.002
236	Thyroid carcinoma	0.055	0.006
237	Tietz syndrome	0.012	0.004
238	Tuberculoid leprosy	0.010	0.002
239	Tuberculosis	0.112	0.006
240	Type 2 diabetes mellitus	0.063	0.025
241	Type II hypersensitivity reaction disease	0.013	0.004
242	Ulcerative colitis	0.635	0.070
243	Urinary bladder carcinoma	0.054	0.021
244	Urticaria	0.080	0.007
245	Uveitis	0.583	0.042
246	Van der Woude syndrome	0.021	0.012
247	Varicocele	0.036	0.018
248	Vascular disease	0.017	0.001
249	Vasculitis	0.045	0.021
250	Venous thromboembolism	0.030	0.013
251	Viral disease	0.044	0.006
252	Visceral leishmaniasis	0.054	0.006

Table S5. List of the human diseases associated with TNF and S100A13 protein, according to Open Targets Platform database (https://platform.opentargets.org/), and corresponding association scores (https://platform-docs.opentargets.org/associations#association-scores).

№	Disease	TNF	S100A13
1	Adenomyosis	0.027	0.001
2	Atherosclerosis	0.043	0.004
3	Biliary atresia	0.041	0.007
4	Blood protein measurement	0.285	0.043
5	Breast cancer	0.103	0.004
6	Cancer	0.113	0.017
7	Cerebral ischemia	0.035	0.002
8	Coronary artery disease	0.114	0.010
9	Cyst	0.044	0.010
10	Diabetic retinopathy	0.040	0.007
11	Duane retraction syndrome	0.051	0.021
12	Endometriosis	0.094	0.014
13	Eosinophilia	0.018	0.001
14	Follicular thyroid carcinoma	0.007	0.003
15	Gonorrhea	0.073	0.002
16	Hippocampal sclerosis of aging	0.082	0.004
17	Hyperglycemia	0.046	0.003
18	Hypertension	0.395	0.004
19	Immature platelet fraction	0.064	0.004
20	Infection	0.120	0.007
21	Interstitial lung disease	0.102	0.007
22	Lung cancer	0.267	0.042
23	Melanoma	0.127	0.032
24	Metastatic malignant neoplasm	0.011	0.002
25	Metastatic melanoma	0.080	0.010
26	Moebius syndrome	0.018	0.005
27	Neoplasm	0.130	0.059
28	Non-small cell lung carcinoma	0.103	0.074
29	Osteogenesis imperfecta	0.019	0.072
30	Osteosarcoma	0.112	0.007
31	Ovarian carcinoma	0.025	0.001
32	Ovarian endometriosis	0.004	0.001
33	Ovarian serous adenocarcinoma	0.008	0.007
34	Pachyonychia congenita	0.033	0.008
35	Papillary thyroid carcinoma	0.084	0.012
36	Pulmonary fibrosis	0.085	0.008
37	Squamous cell lung carcinoma	0.005	0.011
38	Thyroid carcinoma	0.055	0.019
39	Thyroid gland undifferentiated (anaplastic) carcinoma	0.023	0.003
40	Thyroid neoplasm	0.023	0.005
41	Type 2 diabetes mellitus	0.063	0.007
42	Ulnar-mammary syndrome	0.060	0.001

Table S6. List of the human diseases associated with TNF and S100A11/A12/A13 proteins, according to Open Targets Platform database (https://platform.opentargets.org/), and corresponding association scores (https://platform-docs.opentargets.org/associations#association-scores

№	Disease	TNF	S100A11	S100A12	S100A13
1	Breast cancer	0.103	0.009	0.015	0.004
2	Cancer	0.113	0.038	0.016	0.017
3	Coronary artery disease	0.114	0.001	0.041	0.010
4	Gonorrhea	0.073	0.002	0.024	0.002
5	Hyperglycemia	0.046	0.001	0.013	0.003
6	Immature platelet fraction	0.064	0.004	0.002	0.004
7	Infection	0.120	0.018	0.041	0.007
8	Melanoma	0.127	0.002	0.007	0.032
9	Neoplasm	0.130	0.095	0.071	0.059
10	Non-small cell lung carcinoma	0.103	0.079	0.033	0.074
11	Osteosarcoma	0.112	0.025	0.016	0.007
12	Papillary thyroid carcinoma	0.084	0.020	0.076	0.012
13	Type 2 diabetes mellitus	0.063	0.004	0.025	0.007

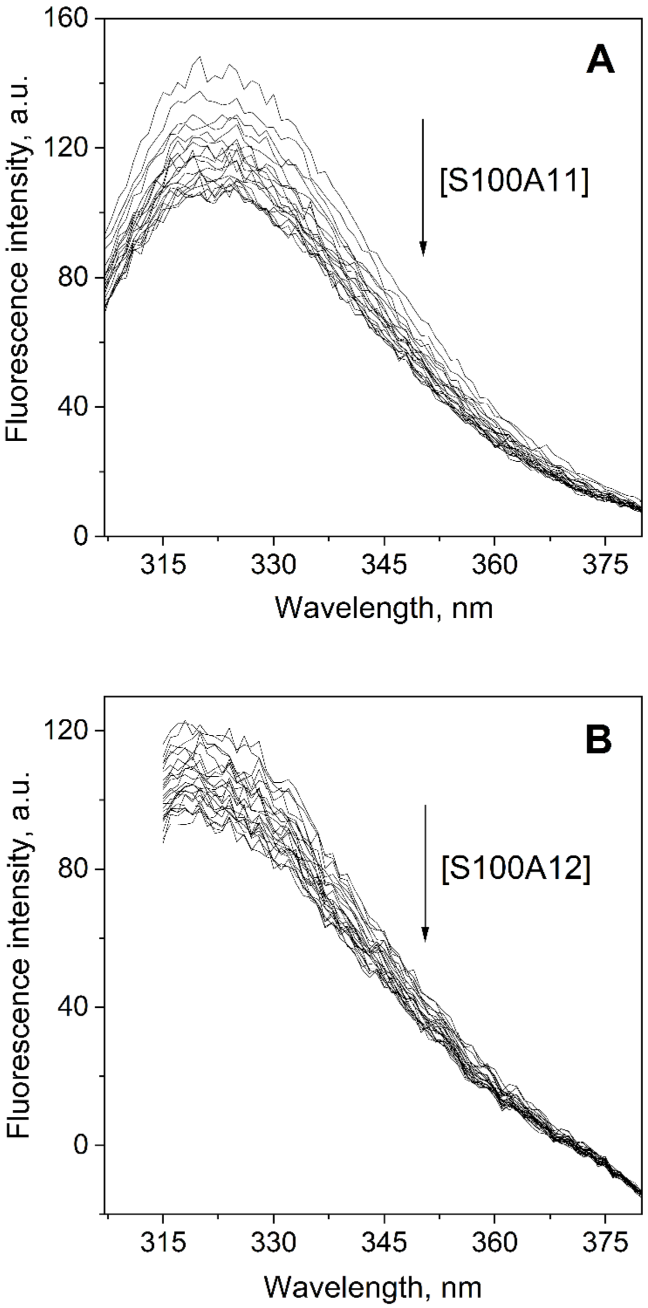

Figure S1. The original spectra of Trp fluorescence of sTNF in the course of its titration with a stock solution of Ca^{2+}-bound S100A11 (panel **A**) or S100A12 (**B**) at 25°C (10 mM HEPES-NaOH, 150 mM NaCl, 1 mM $CaCl_2$, pH 7.4). sTNF concentration of 280 nM or 0.5 µM for the titrations by S100A11 and S100A12, respectively. The excitation wavelength was 295/298 nm. The spectra were corrected for spectral sensitivity of the fluorimeter [1]. The arrows indicate the direction of increasing concentration of the S100 protein.

References

1. Burstein, E. A.; Emelyanenko, V. I., Log-normal description of fluorescence spectra of organic fluorophores. *Photocem.Photobiol.* **1996,** *64*, 316-320.
